# Pre-protocol of the Virtual Spatial Configuration Task (VSCT): A Novel Virtual Reality-Based Tool for Assessing Cognitive Map Formation Abilities

**DOI:** 10.12688/openreseurope.19436.3

**Published:** 2025-10-31

**Authors:** Alberto Massimiliano Umiltà, Giorgio Li Pira, Ford Burles, Giovanni Ottoboni, Alessia Tessari

**Affiliations:** 1 Psicologia, Universita degli Studi di Bologna Dipartimento di Psicologia Renzo Canestrari, Bologna, Emilia-Romagna, 40126, Italy; 2 Psychiatry, University of Calgary Department of Psychiatry, Calgary, Alberta, Canada

**Keywords:** spatial cognition, recall, working memory, cognitive neuroscience

## Abstract

This study proposes the validation of the Virtual Spatial Configuration Task (VSCT), a novel task designed to evaluate cognitive map formation abilities in participants. Addressing a notable gap in spatial cognition research, particularly in the assessment of higher-level spatial abilities in 3D environments, the VSCT offers a virtual reality (VR) approach that allows users to explore and recall spatial relationships between landmarks. This task is particularly innovative for populations with impaired mobility, as it simplifies navigation by restricting movement to rotational exploration, thus improving accessibility and reducing motion sickness. Furthermore, the VSCT could serve a dual purpose: assessing spatial orientation skills while also providing a platform for training and improving these skills post-injury. The potential applications of this tool extend to neurorehabilitation and other therapeutic interventions, offering an engaging, immersive method for enhancing spatial abilities.

## Introduction

“Spatial ability can be defined as the capacity to generate, retain, retrieve and transform spatial representations” ( Burles, 2014 pp.4). Those abilities depend on multiple sensory cues, computational mechanisms, and spatial representations ( Umiltà, 2021). The efficacy of spatial orientation and navigation depends on the complex integration and manipulation of multisensory information across time and space. Spatial abilities in humans have been investigated using two types of tasks: figural-scale and environmental-scale ( Jansen, 2009). Figural-scale tasks assess abilities such as spatial perception, mental rotation, visualization, and orientation ( Linn & Petersen, 1985), and environmental-scale tasks involve large stimuli like rooms, buildings, and neighborhoods ( Hegarty *et al*., 2006).

As pointed out by Bartlett and Camba (2022), spatial ability tests that attempt to assess 3D abilities through 2D media have limitations: Any 2D image is inherently ambiguous when it comes to interpreting a 3D shape from the image ( Pizlo, 2008). Black and white line drawings used in these tests introduce ambiguity due to perceptual challenges such as multi-stability and difficulties associated with axonometric projections, which do not align naturally with human visual perception and can be complex to interpret Bartlett & Camba (2022). Furthermore, 2D tests do not allow participants to move and explore the environment. This factor is crucial considering the role of proprioceptive feedback and the vestibular system in the encoding and maintenance of spatial configurations in memory. The lack of proprioceptive feedback means participants are unable to use the sensory information from their own movements and body positions to build and maintain accurate spatial representations. This omission neglects the dynamic and interactive nature of spatial learning and navigation, where proprioceptive cues significantly enhance the fidelity of the cognitive map by providing continuous updates about one's position and movement within the environment. ( Bayramova *et al*., 2021; Fetsch *et al*., 2009; Nardini *et al*., 2008). Here we define cognitive map as a mental representation of the spatial layout of an environment, allowing an individual to acquire, code, store, recall, and decode information about the relative locations of places, landmarks, and routes ( Tolman, 1948).

In our study, translational movement is not performed by participants. Instead, participants are seated in a swivel chair that allows them to actively rotate their torso left and right, enabling 360-degree exploration of the environment. These self-generated rotations provide limited but present vestibular and proprioceptive feedback. In particular, yaw-plane movements engage the vestibular system via stimulation of the semicircular canals responsible for detecting angular acceleration ( Ehtemam *et al.*, 2012), while proprioceptive input is involved through trunk muscle activity and postural adjustments during chair rotation ( Ivanenko *et al.*, 1999).

Moreover, vestibular signal processing during active rotation is known to depend on the match between predicted and actual proprioceptive feedback. When this match is present, the brain can suppress vestibular reafference, allowing for stable perception of self-motion. However, in the presence of a mismatch, vestibular neurons continue to encode the full signal, underscoring the functional role of proprioceptive input even in non-translational movement contexts ( Brooks & Cullen, 2014); ( Cullen & Zobeiri, 2021).

### Strategies of spatial orientation and navigation 

Large-scale spatial cognition involves complex processes such as learning spatial relationships between environmental objects, maintaining these relationships in memory, and using this knowledge to navigate and communicate spatial information ( Hegarty *et al.*, 2006). Vision is typically the primary sense for understanding spatial layouts, but non-visual senses also play critical roles, including the vestibular system, which informs about linear and angular accelerations ( Casale *et al.*, 2023), kinesthesis, which senses limb movement ( Ehinger *et al.*, 2014), and proprioceptive information about self-movement and body position originates from sensors in our muscles and joints ( Chance *et al.*, 1998; Sheeran & Ahmed, 2020). Working memory helps piece together spaces from multiple perspectives, encoding information in various formats such as verbal sequences of route directions or spatial configurations ( Hegarty *et al.*, 2006). Research shows that encoding self-motion through body-based senses enhances the ability to create and use cognitive maps effectively ( Bayramova *et al.*, 2021). Two types of mental representation of spatial layouts are typically distinguished: Route Knowledge, which consists of rigid, egocentric representations based on sequential routes and landmarks ( Aguirre & D’Esposito, 1999), and Cognitive Maps, which are flexible, allocentric representations storing the identities and spatial relationships of landmarks ( Aguirre & D’Esposito, 1999; Tolman, 1948). This strategy allows navigation using landmarks and their relative positions ( O’keefe & Nadel, 1979; Tolman, 1948). Moreover, spatial navigation can rely on allocentric (approaches where an individual or organism navigates the environment using an external frame of reference, independent of their own position or orientation) and egocentric (approaches where an individual or organism navigates the environment based on their own position and orientation) strategies, and the distinction isn't always straightforward, often depending on individual differences, as well as task and environmental demands. The most reliable source of information dynamically determines the navigation approach, although disorientation can still occur in novel environments, highlighting the complexity of spatial navigation ( Fetsch *et al.*, 2009; Nardini *et al.*, 2008). Environmental factors play a crucial role here; in landmark-rich environments, allocentric strategies tend to dominate, whereas featureless environments like deserts may rely more on egocentric cues, despite their potential for error and disorientation ( Souman *et al.*, 2009). Therefore, while allocentric and egocentric strategies provide foundational frameworks for understanding spatial cognition, navigation involves a complex interplay of these strategies and their integration with various cognitive processes. These factors must be considered in assessing spatial abilities

### Validation of a new 3D spatial task

In spatial cognition research, there is a notable gap concerning the development of programs designed to assess higher-level spatial abilities, such as the formation of cognitive maps in 3D environments.

Research has shown that individual differences in working memory and navigational strategies significantly impact the ability to form cognitive maps, with some individuals relying more on route-based navigation while others are better at forming place-based, map-like representations ( Weisberg & Newcombe, 2016).

Efforts to improve assessment performance, such as clarifying drawings, enhancing 3D appearance, or using physical models and animations, have shown positive results ( Cohen & Hegarty, 2012; Fisher *et al*., 2018; Yue, 2008). For instance, the "Spatial Configuration Task" (SCT, Burles *et al.*, 2017) has been designed to investigate the generation of cognitive maps in a simple 3D Virtual Environment (VE). In this task, participants first become familiar with a space where objects are arranged for memorization. This task was designed to provide a quick and reliable measure of the ability to generate and utilize a mental representation of a simple virtual environment (VE), but it has never been implemented in an immersive virtual reality (VR) environment. 

However, these modifications have not yet been widely adopted in the evaluation of cognitive map formation abilities ( Bartlett & Camba, 2022). VR offers several advantages over traditional 2D and desktop 3D assessments of spatial abilities, including the ability to evaluate 3D maps in a fully immersive 3D environment. Additionally, VR can incorporate sensory information related to body movement, providing an additional source of input for cognitive map formation, thereby enabling a more ecological and realistic assessment. Therefore, we propose a protocol for validating a novel VR task, the Virtual Spatial Cognition Task (VSCT). This task simulates participants' cognitive map formation abilities by requiring them to explore a virtual environment, learn spatial relationships between landmarks, and recall their positions.

Developing a spatial task that allows users to explore the environment by rotating in a movable chair without the need for forward or backward movement not only leverages several benefits associated with gaze-directed steering (GDS) but also simplifies interaction by eliminating the need for complex movement controls, thus making VR more accessible, especially for users with limited mobility or dexterity.

 A significant advantage expected to feature this setup is represented by the reduced motion sickness. By restricting navigation to rotational exploration should avoid the sensory mismatch that often causes discomfort and enhances situational awareness, allowing users to freely observe their surroundings without the distraction of forward or backward movement.

Moreover, the design controls the visibility of virtual objects, requiring users to piece together information gradually, a feature shared also with the SCT.

This study aims to validate the newly developed VSCT by evaluating participants' performance on the original SCT both before and after VR exposure. We will use a **between-subjects design**, dividing participants into two groups to clarify the specific effects of spatial versus non-spatial cognitive demands. One group will perform the task in virtual reality, while the other will engage in a non-spatial auditory verbal task, adapted from Bacon *et al.* (2008), to isolate memory processes from spatial reasoning. To this end, only the first administration of the SCT (T1) will be used for correlation analyses with the VSCT, as it provides a baseline measure of spatial performance. The second administration (T2) is included solely for exploratory purposes—to investigate whether repeated exposure might lead to performance improvements and to preliminarily explore potential training effects. These exploratory findings could inform the design of future studies specifically aimed at assessing the impact of training or interventions. Establishing the VSCT’s validity is a necessary first step before we can confidently use it in such longitudinal or interventional contexts.

We will use a series of tests and tasks to validate the VSCT. We hypothesize a positive correlation between the VSCT and SCT, as both are designed to measure the same construct—namely, the ability to form cognitive maps. We also hypothesize a positive correlation between the VSCT and Backward Corsi, as working memory is a key component in the formation and maintenance of cognitive maps ( Epstein *et al.*, 2017). Conversely, we do not expect a significant correlation between the VSCT and RAVLT. We included the Rey Auditory Verbal Learning Test (RAVLT) in our study precisely to assess discriminant validity. The RAVLT measures verbal episodic memory, while the VSCT targets visuospatial configuration memory—two constructs that are theoretically and empirically distinct ( Suri *et al.*, 2017). By including the RAVLT and confirming that it does not significantly correlate with performance on the VSCT, we provide evidence that the VSCT is not simply capturing general memory ability, but rather a more specific visuospatial memory component. This lack of correlation supports the discriminant validity of our task, helping to confirm that it measures a construct different from that assessed by standard verbal memory tasks.

Finally, to evaluate VSCT feasibility we will use the Simulator Sickness Questionnaire (SSQ) ( Kennedy *et al*., 1993).

## Methods

### Open science commitment

Our protocol is available on the Open Science Network, and our data will be made accessible in the near future ( https://osf.io/3dmsy/).

### Participants

As the study aims to evaluate **convergent and discriminant validity** of VSCT, by comparing VSCT correlation with SCT to the correlation of RAVLT with SCT, an *a priori* power analysis was conducted using G*Power 3.1 Faul *et al.* (2009). Assuming a **moderate to large effect size (Cohen’s q = 0.4)**, a significance level of **α = 0.05**, and a desired power of **0.95**, The required sample size for comparing two independent correlation coefficients using a z-test for dependent Pearson’s r was calculated as 166 **participants**. Taken this for granted, and bearing in mind that the data to correlate are not yielded with a paper-and-pencil questionnaire- but a virtual setting requiring training and adaptation, we weight the effect size indication with previous research using similar aims and methods.

This was the case in da Costa *et al.* (2022), who validated two immersive virtual reality tasks for spatial orientation using correlational and group comparison analyses. The authors reported significant associations between the virtual tasks and traditional cognitive measures, as well as successful group discrimination.

The participants represented a sample size large enough to yield statistically reliable confidence intervals. It should be noted that the study employed a between-subjects design; however, participants from the two groups were combined. In contrast, our study will correlate the two groups with two different variables.

Furthermore, *He *et al.* (2022)* employed a fully within-subjects design to validate a spatial perspective-taking task in ambulatory virtual reality, using a sample size comparable to ours. However, since within-subjects designs typically offer greater statistical power, we acknowledge that our between-subjects design may require a larger sample to achieve equivalent sensitivity. We have accounted for this by adopting a conservative effect size estimate and grounding our decision in the specific demands of VR-based testing, which introduces variability due to training and adaptation phases.

Participants will be actively recruited to participate in the intervention across various University of Bologna Campus locations. Recruitment will be facilitated through targeted advertisements and consecutive enrollment procedures to ensure diverse participant representation and data robustness. 

The project will be advertised in public spaces and social platforms. Participants will be invited to make an appointment with the researchers by e-mail after the first evaluation phase. During this initial contact, the researchers will explain the purpose of the study, answer any follow-up questions, and invite participants to come to the Department of Psychology. Upon arrival at the lab, participants will be asked to sign an informed consent form and compile the personal data processing form. No identifying personal information (e.g., names, contact details, or video recordings) will be collected or stored. Participants are identified only by randomized numeric codes, and all reported results will be presented at the group level, ensuring that no individual can be identified in any publication or presentation of the data.

### Informed consent

Informed consent will be obtained from all participants before participation, in accordance with The Code of Ethics of the World Medical Association (Declaration of Helsinki) (1964). The Bioethics Committee operating within the University of Bologna raised no concern about the study (n. 0022813 dated 2024.01.06).

### Materials

***Instruments***

Individuals who agree to participate will be assessed online through a link to the questionnaires (via Psytoolkit and Qualtrics platform). The first evaluation will be carried out using an ad-hoc form to collect sociodemographic data and the following psychometric tests. 

*Santa Barbara Sense of Direction Scale* (SBSOD): The SBSOD assesses a participant’s self-reported sense of direction. The scale consists of 15 orientation-related statements (e.g. ‘I am very good at giving directions’) that participants rate on a seven-point Likert scale ranging from Strongly Agree (1) to Strongly Disagree (7). The SBSOD significantly correlates with measures of large-scale spatial skills in numerous experiments ( Hegarty *et al*., 2002; Ventura *et al*., 2013).

*Backward Corsi Task*: The Backward Corsi Task ( Isaacs & Vargha-Khadem, 1989; Kessels *et al*., 2008) is a cognitive tool designed to evaluate visuospatial working memory and attention by requiring participants to replicate sequences of block taps in reverse order.

The spatial performance of each participant will be measured using the following tasks.

*Spatial Configuration Task* (SCT): The Spatial Configuration Task is a quick and reliable measure of one's ability to generate and use a mental representation of the spatial configuration of different objects in a 2D virtual environment. ( Burles *et al*., 2017)

*The Rey Auditory Verbal Learning Test* (RAVLT) The Rey Auditory Verbal Learning Test (RAVLT) is a widely used neuropsychological assessment that measures verbal memory and learning. In this test, participants are presented with a list of 15 words, which they must recall across multiple trials. After a delay, they are asked to recall the list again and later recognize the words from a larger list of distractor words. The test assesses various memory functions, including immediate recall, learning ability, delayed recall, and recognition memory, helping evaluate cognitive functions related to verbal learning and memory ( Peaker & Stewart, 1989).

At the end of VSCT the SSQ will be administered to assess the feasibility of the task. The SSQ is a tool used to assess the severity and types of discomfort or sickness symptoms that individuals experience when exposed to virtual environments, simulators, or similar immersive technologies. It consists of a self-report questionnaire where individuals rate the severity of various symptoms on a 4-point scale (0 = None, 1 = Slight, 2 = Moderate, 3 = Severe) ( Kennedy *et al*., 1993).

### *Virtual Spatial Configuration Task* VSCT

Hardware: The task is displayed via an HTC Vive Pro Eye (display resolution of 1440 × 1600 pixels per eye and 90 Hz refresh rate) head-mounted dis-play (HMD). The HMD is equipped with an eye tracking system running at 120 Hz sampling rate and reported to achieve a spatial accuracy of 0.5° - 1.1° ( HTC Corporation, 2021; VIVE European Union (n.d.)). Each participant is tested using the following lab setups: two SteamVR base stations ("lighthouses"; Valve Corp., Bellevue, WA,USA) are set up in the lab room for positional tracking, the lab is equipped with a VR-capable desktop computer (AMD Ryzen 9 5900HX CPU, 3.30GHz, 32GB RAM, AMD Radeon™ RX 6600M GPU). 

Software. The experiment is implemented in Unity using the Editor Version (version 2023.11f1).

***Scene setup***

To design the virtual environment, we created an empty space and selected 5 objects (a chair, a trashcan, a washer, a lamp and a plant). We chose objects that could be easily found in the house but from different rooms; this choice was made to avoid semantic facilitation or cueing in localizing the object (e.g. The table should be near the kitchen and so on) ( Lu *et al*., 2024).

Furthermore, we opted to use familiar household objects rather than the geometrical shapes traditionally employed in the Spatial Configuration Test (SCT). This decision was motivated by the unique capabilities of virtual reality, which enable the creation of immersive, ecologically valid scenarios. By selecting objects with high familiarity and recognizability, we aimed to reduce variability in object encoding and promote more consistent performance across participants. Additionally, projecting participants into environments that mirror real-life contexts—such as recognizing and remembering the arrangement of common objects within a room—enhanced both the ecological validity of the task and participant engagement.

After objects selection, the floor and the lighting are meticulously crafted. To ensure that no spatial cues such as sun position or shadow direction are given to the participant, we placed the sun directly above the head of the participant, selected “no shadows” as the shadow type for each object, and chose a uniform smooth grey floor. The objects were placed in a circular arrangement with equidistant spacing.

We use the created scene as a template for 10 different scenes, 5 dedicated to the training phase and 5 dedicated to the testing phase. 

***The virtual task***

**Figure 1.**
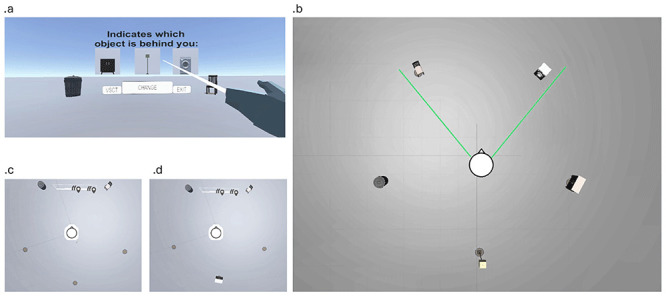
**a.** Main menu interface presented at the beginning of each scene, allowing participants to change scenes, return to the home screen, or exit the task. **b.** Example view of the full environment, showing all five objects placed in a triangular configuration. This layout illustrates the type of spatial arrangement participants are required to learn. **c.** At the start of each trial, only the two front objects are visible to the participant, who must respond to a question about the missing (third) object based on memory. A floor circle indicates the location of the hidden object. **d.** If the participant selects the wrong answer, the environment allows them to rotate and discover the third object's location. This feedback mechanism supports gradual learning of the spatial configuration.

We selected three objects for each scene: two placed near each other and a third positioned on the opposite side, facing the pair. The participant's camera was placed in front of the two adjacent objects, such that the third object was located behind them, forming a triangular spatial configuration ( Figure 1b).

The location of the missing object was indicated by a circle on the floor ( Figure 1c/d), providing a visual landmark to aid participants. An interactive interface allowed participants to switch scenes by pressing "change", return to the main menu with "VSCT", and exit by pressing "exit" ( Figure 1a). Participants could explore objects around them for a maximum of 20 seconds per scene.

From the beginning of the task, participants were prompted with questions about the spatial location of objects. Each scene included a canvas displaying a question (e.g., "Which object is behind you?") with three possible choices representing the missing object. Only the two adjacent objects in front of the participant were shown ( Figure 1b). Button positions were randomized to avoid spatial response biases. Participants learned the correct answers through trial and error, gradually forming internal representations of the spatial configurations based on feedback. The task consisted of 40 such trials. For each trial, the participant’s response was recorded.

***Environment exploration***

Participants could rotate and inspect the environment by moving right or left, using the Unity Interaction Toolkit and custom scripts to interface with the HMD joysticks. Users’ movements are continuous and based on the participant's head direction to ensure ecological validity with proprioceptive feedback and reduce motion sickness.

***Data repository***

At the end of the project, the project source code will be published in a dedicated public repository on Git Hub to encourage further development and research ( https://github.com/gio-lp/VSCT). 

***Procedure***

**Figure 2.**
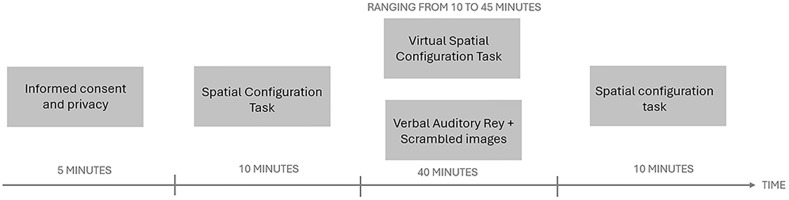
Figure depicting the timelie of the experimental procedure.

 Participants will be assigned in a pseudo-randomized single-blinded manner to one of the two groups (Auditory Verbal (AV) vs. Virtual Reality (VR)). Both groups will be asked to perform an initial assessment of their spatial abilities using the 40-item version of SCT. In the next phase, the experimental group will be made to wear the "HTC VIVE" visor that will allow them to perform the Virtual Spatial Configuration Task in VR (VR condition); the control group will be administered the auditory verbal memory task (AV condition). Both groups will be given the SCT again at the end of the task (See Figure 2). 

***VR spatial task condition***

 Training: The participants will be placed in our virtual environment and instructed to explore the space around them in each of the scenes. In each scene, the participant will see 3 objects at a time (two in front of him and one behind him).

Testing: After the training is completed, participants will be asked to start the testing phase. This phase will last until the task is completed, namely, when the participant accurately recalls the position of the objects after three consecutive correct trials. The objects are those learned during the training phase. The participants' accuracy and reaction time will be recorded.

***AV memory task condition***

Administration of the RAVLT will involve several steps:

The experimenter will read the first list (List A) of 15 unrelated words at a rate of about one word per second. It's crucial to present the words in the same order and with the same pronunciation for each participant. After presenting the list, the participant will be asked to repeat back as many words as they can remember, regardless of the order. After the participant recalled as many words as possible from List A, the process will be repeated for five immediate recall trials. After each trial, the number of words correctly recalled will be recorded. 

The experimenter will read a second list (List A) of 15 unrelated words (List B) to the participant and instruct them to recall as many words as possible from this list. This step is designed to interfere with the participant's ability to recall words from List A. Following the presentation of List B, the participant will be asked to recall the words from List A once again. This step assesses the individual's ability to retrieve information from memory after interference.

After a short delay in which the participant will have to do a scrambled image test (usually around 20–30 minutes), the participant will be asked to recall the words from List A once more without presenting the word list again. This step evaluates the individual's ability to retain information in memory over time.

The participant's performance will be scored based on the number of words correctly recalled or recognized during each test phase.

## Plan of analysis

### Independent samples t-test

An independent samples t-test will be conducted to compare the average number of hits at the SCT between the control and experimental groups. Age and sex were collected as sociodemographic variables because both have been shown to influence spatial navigation abilities in previous research ( Sneider *et al.*, 2015). Although we did not formulate specific hypotheses regarding the effects of age or sex, these variables were included to confirm group equivalence and to control for potential confounding factors, thereby strengthening the internal validity of our findings. Moreover, to verify that the two groups do not differ significantly in these scores, independent samples t-tests will be conducted to compare the CORSI and SBSOD scores between the experimental and control groups.

### Correlation analysis

We will run correlations matrix between CORSI, SBSOD, and hits at both the VSCT and SCT. This will help us understand the influence of general intelligence and working memory capacity on spatial ability and ensure that the VSCT and SCT are comparable tasks

Regarding reliability, we plan to assess internal consistency using split-half and/or Cronbach’s alpha methods for key performance metrics (e.g., accuracy, error scores), depending on distributional characteristics. In addition, we aim to conduct a test–retest reliability study in a subsample of participants to examine stability over time.

### Expected results

***Validation of VSCT outcomes***

We expect SCT mean accuracy to be positively correlated with performance on the CORSI backward task and VSCT accuracy.

A positive correlation with SCT accuracy would validate VSCT as a measure of participants' abilities to maintain and retrieve cognitive maps based on allocentric frameworks ( Burles *et al*., 2017). Both SCT and VSCT require creating an allocentric cognitive map from multiple egocentric viewpoints. Therefore, we expect a positive correlation between SCT mean accuracy and spatial working memory, as measured by the Corsi backward task. Regarding reliability, based on our planned analyses, we expect to find acceptable to high internal consistency for key performance metrics (e.g., accuracy, error scores), as assessed through split-half reliability and/or Cronbach’s alpha, depending on the distributional properties of the data. Additionally, we anticipate that test–retest reliability in a subsample will demonstrate moderate to high stability over time, supporting the robustness of both the SCT and VSCT as reliable tools for assessing spatial cognition.

## Discussion

The proposed study protocol aims to validate a novel Virtual Spatial Configuration Task (VSCT), designed to evaluate participants' cognitive map formation abilities. This validation is crucial as it establishes the reliability of the VSCT in assessing spatial orientation skills. By requiring participants to explore a virtual environment, learn spatial relationships between landmarks, and recall their positions, the task offers a comprehensive measure of spatial cognition.

The development of the VSCT addresses a significant gap in spatial cognition research, particularly the lack of programs designed to assess higher-level spatial abilities, such as cognitive map formation, in 3D VR environments. Although some research groups have proposed VR-based spatial tests (e.g., Gehrke *et al*., 2018; Hartman *et al*., 2006), to the authors' knowledge, no specific validations of VR assessment tasks in immersive virtual environments have been conducted. Therefore, we believe it is important to address this gap and explore the opportunities that VR offers in the domain of spatial cognition assessment.

One of the innovative aspects of the VSCT is its potential application for populations with impaired movement. The task is designed to allow users to explore the environment by rotating in a movable chair, eliminating the need for forward or backward movement, making it particularly suitable for individuals with limited mobility or dexterity. This design simplifies interaction, reduces the complexity of movement controls, and enhances accessibility. Additionally, by limiting navigation to rotational exploration, the task minimizes the sensory mismatch that often leads to motion sickness, thereby improving user comfort and engagement. The VSCT's design strategically controls the visibility of virtual objects, requiring users to piece together information gradually. This approach encourages cognitive engagement and problem-solving as users integrate sequential observations to gain a comprehensive understanding of the environment. Such cognitive engagement is particularly beneficial for populations with impaired movement, as it provides a means to enhance spatial orientation and navigation skills without the need for physical locomotion. For instance, stroke survivors often experience limited mobility and spatial disorientation. The VSCT’s rotation-based exploration allows them to safely practice navigating a virtual environment. By gradually revealing objects as they turn, users strengthen spatial awareness and problem-solving skills without the need for walking or complex controls.

The task's reliance on proprioceptive feedback and vestibular system inputs significantly enhances its effectiveness in spatial learning and navigation. Unlike static 2D tasks, which fail to provide participants with sensory information from their own movements, this dynamic approach enables users to actively explore and interact with the environment. By integrating proprioceptive cues and vestibular inputs, participants receive continuous updates about their position and movements, allowing them to build and maintain accurate cognitive maps. This interactive, multi-sensory experience reflects the real-world processes of spatial encoding and navigation, underscoring the critical role of proprioceptive and vestibular feedback in enhancing the fidelity and robustness of spatial representations. In this context, it is also important to consider the applicability of this software for training spatial abilities after injury, which often involves some level of motor impairment. While previous studies have demonstrated the malleability of lower-level spatial functions, such as mental rotation and route memory, through various training interventions ( Montana *et al*., 2019; Subrahmanyam & Greenfield, 1994; Uttal *et al*., 2013), few have specifically targeted cognitive map formation. An exception is a study by McLaren-Gradinaru and colleagues (2020), which evaluated the effects of training by comparing task performance before and after training. By providing an immersive virtual environment where participants can develop and refine their spatial orientation skills, the VSCT could serve the dual purpose of assessing and improving the ability to form cognitive maps.

The translational value of the VSCT extends beyond its immediate application in cognitive training and neurorehabilitation. By providing a validated tool for assessing and improving spatial orientation skills, the VSCT has the potential to be integrated into a wide range of therapeutic and educational programs. For individuals with neurodegenerative diseases, brain injuries, or developmental disorders, the VSCT offers a novel and engaging way to enhance spatial cognition and independence. Its use in virtual reality settings also opens up possibilities for remote and home-based interventions, making it a versatile tool for widespread application.

Beyond clinical populations, the VSCT could also hold significant value for broader groups at risk of spatial difficulties. For example, healthy older adults often experience age-related declines in spatial orientation that increase the risk of disorientation and falls; the VSCT may provide an engaging and safe way to monitor and train these abilities proactively. Similarly, in educational contexts, spatial ability is closely linked to achievement in STEM disciplines. Incorporating the VSCT into training curricula could provide students with opportunities to strengthen cognitive map formation skills that underlie problem-solving in fields such as mathematics, engineering, and the natural sciences. By extending its scope to these populations, the VSCT not only serves as a rehabilitative and diagnostic tool but also as a platform with the potential to foster resilience, independence, and academic success.

Looking to the future, the VSCT could be further refined and expanded to include more complex virtual environments and tasks. Integrating advanced features such as adaptive difficulty levels, real-time feedback, and multi-sensory integration could enhance its effectiveness and user experience. Additionally, research could explore the long-term effects of regular VSCT training on spatial cognition and daily navigation skills, providing valuable insights into its therapeutic potential. Future work could also compare chair-based and ambulatory VR setups to further assess the generalizability of findings across different navigation modalities.

## Conclusion

In conclusion, the validation of the VSCT represents a significant advancement in spatial cognition research. By providing an accessible, engaging, and effective tool for assessing and training spatial orientation skills, the VSCT holds promise for improving the quality of life for individuals with impaired movement and other cognitive challenges. Its translational value lies in its potential to be integrated into diverse therapeutic and educational programs, paving the way for innovative interventions and future research in the field.

## Declarations

### Ethics approval

The study was approved by the Bioethics Committee of the University of Bologna (approval number: n 0022813, dated 2024.01.06) and complies with the ethical standards outlined in the Declaration of Helsinki (1964).

### Consent to participate

Written informed consent will be obtained from all participants involved in this study. Ethical approval has already been granted by the Bioethics Committee of the University of Bologna under Protocol No. [0022813].

Participants will be provided with a detailed consent form outlining the study's objectives, procedures, potential risks, and their rights, including the right to withdraw at any time without consequences.

Furthermore, the full text of the informed consent document will be made available in the Extended Data to ensure transparency and adherence to ethical guidelines.

### Consent for publication

Not applicable, as this is a study protocol and does not include individual data or identifiable information.

## Data Availability

No data associated with this article. The data from the future experiment will be made available in accordance with open science principles. The research protocol has been drafted in the OSF repository with the following DOI: https://doi.org/10.17605/OSF.IO/3DMSY ( Umiltà *et al*., 2024). All the materials, including study questionnaires, informed consent and supplementary data, are also published at this DOI. ***Code availability*** At the end of the project, the project source code will be published in a dedicated public repository on Git Hub to encourage further development and research ( https://github.com/gio-lp/VSCT). Data are available under the terms of the Creative Commons Attribution 4.0 International license (CC-BY 4.0) ( https://creativecommons.org/licenses/by/4.0/).
