# Supplementary material for: Pre-protocol of the Virtual Spatial Configuration Task (VSCT): A Novel Virtual Reality-Based Tool for Assessing Cognitive Map Formation Abilities
Source: Open Res Eur. 2025 Oct 31;5:42. Originally published 2025 Feb 5. [Version 3] doi: 10.12688/openreseurope.19436.3 (PMC12501587; doi:10.12688/openreseurope.19436.3)
Supplement: Peer review history

# Reviewer report: Reviewer response for version 3

**DOI:** 10.21956/openreseurope.23299.r63207

**Published:** 2025-11-06

**Authors:** Jozsef Katona

**Affiliations:** 1 University of Dunaujvaros, Dunaujvaros, Hungary; 2 Óbuda University Kálmán Kandó Faculty of Electrical Engineering (Ringgold ID: 448998), Budapest, Budapest, Hungary

I accept the paper in the current form.

Is the study design appropriate for the research question?

Partly

Is the rationale for, and objectives of, the study clearly described?

Partly

Are sufficient details of the methods provided to allow replication by others?

Yes

Are the datasets clearly presented in a useable and accessible format?

Not applicable

Reviewer Expertise:

Human-computer interaction; cognitive psychology; software engineering.

I confirm that I have read this submission and believe that I have an appropriate level of expertise to confirm that it is of an acceptable scientific standard.

# Reviewer report: Reviewer response for version 3

**DOI:** 10.21956/openreseurope.23299.r63203

**Published:** 2025-11-06

**Authors:** Alastair D. Smith

**Affiliations:** 1 University of Plymouth, Plymouth, UK

The authors have broadly addressed my previous comments adequately. If I were honest, I would still prefer a little more on the original SCT (so that one can get a better grasp of the similarities and differences between the variants, and how performance has previously related to other measures) and perhaps a little less on the multiplicity of potential applications of the VSCT if successfully validated. However, I am but one voice, and the overarching aims and objectives seem fine to me.

Is the study design appropriate for the research question?

Partly

Is the rationale for, and objectives of, the study clearly described?

Partly

Are sufficient details of the methods provided to allow replication by others?

No

Are the datasets clearly presented in a useable and accessible format?

Not applicable

Reviewer Expertise:

Spatial cognition

I confirm that I have read this submission and believe that I have an appropriate level of expertise to confirm that it is of an acceptable scientific standard.

# Reviewer report: Reviewer response for version 2

**DOI:** 10.21956/openreseurope.21974.r54390

**Published:** 2025-10-06

**Authors:** Laura Miola

**Affiliations:** 1 University of Padova, Padua, Italy

I would like to thank the authors for their responses to my questions and concerns. The clarifications provided have been valuable, and I think that this revised protocol can be considered in the next stage of the process.

Is the study design appropriate for the research question?

Partly

Is the rationale for, and objectives of, the study clearly described?

Yes

Are sufficient details of the methods provided to allow replication by others?

Partly

Are the datasets clearly presented in a useable and accessible format?

Not applicable

Reviewer Expertise:

Spatial cognition, individual differences, environmental psychology

I confirm that I have read this submission and believe that I have an appropriate level of expertise to confirm that it is of an acceptable scientific standard.

# Reviewer report: Reviewer response for version 2

**DOI:** 10.21956/openreseurope.21974.r60516

**Published:** 2025-10-01

**Authors:** Meytal Wilf

**Affiliations:** 1 Weizmann Institute of Science, Rehovot, Israel

Open Science Europe review:

 The authors present a proposal for a virtual adaptation of the ‘spatial configuration task’. The new paradigm will present a more immersive and ecological measure of spatial cognition, with potential implementations for persons with restricted mobility. The protocol aims to validate their new paradigm on a group of healthy individuals and compare it to more conventional tests for spatial cognition. Although the idea and paradigm are solid and potentially useful, I have comments about the design and about the clarity and organization of the text. I detail my comments below, according to the chronological order of the different sections:

 Abstract:

- Why do the authors assume that rotational movement will cause less cyber sickness the other types of locomotion? Is this corroborated by previous studies?

Introduction:

- Still missing in the description of the standard SCT – what is the task participants need to perform after memorizing the objects?
- Also please clarify - Is the conventional SCT 2D or 3D (or maybe a 3D simulated on a 3D screen)? From the description in the introduction, it appears that it is already 3D and virtual. If so, not clear what immersive VR adds to this.
- Relatedly – the authors list “the ability to evaluate 3D maps in a fully immersive 3D environment” as one of the benefits of VR. Please write it more specifically – does that refer to the added depth dimension? The isolation from the external environment?  A switch from allocentric to egocentric perspective?
- The authors refer to SCT as ‘spatial configuration task’, and then the VSCT is referred to as ‘virtual Spatial Cognition Task’. If it is a VR version of the same task, I suggest keeping the same naming, as it is also more specific (i.e., ‘virtual spatial configuration task’).
- Similarly to the abstract – will rotational movements cause less dizziness than actual locomotion? Are there studies to corroborate this assumption? If there is no delay between the movement and the change in visual inputs (like is the case in most new VR devices), then no extra motion sickness is expected by walking. It might be that rotating the head is causing more risk of VR-related motion sickness.
- The authors state the ‘the design controls the visibility of virtual objects’, as in the SCT. Please either clarify how this is implemented (also in the SCT), or move this sentence to the methods after the detailed description of the task.
- Last paragraph in page 4 – from the first sentence it is not clear what is the goal of running the SCT pre and post the VSCT. Why is it important for validation? Are there learning processes expected? Although it is outlined at the end of the paragraph, the first sentence should be rephrased for consistency (as only the pre SCT is used for the validation).
- Please describe in a few words the auditory verbal task in the same paragraph – does it have a memory component?
- In the last paragraph of the introduction the authors lay out their predications. Since there are readers who might not be familiar with the Corsi and the RAVLT tasks, please add a short description of each of the tasks before laying out the predictions.
- Also related to predictions – why do the authors assume there will be no correlation between VSCT and RAVLT? From what I understand in their description of the task, participants will be asked to recall a list of objects in VSCT, which requires some similar capabilities as those utilized in RAVLT.

Methods:

- In the participants section, the authors write that “Participants will be actively recruited to participate in the intervention”. This proposed study is a validation and not an intervention.
- When describing SCT, please outline what is the task exactly, and not only what is its purpose. Fully understanding this task is central to understanding the study.
- In the ‘scene setup’ section, how were the 10 different scenes different from each other? By the identity of the objects? By their location? This is also the first time that the authors state there is a training phase and a testing phase. At this point it is not clear why training is needed for the validation study.
- ‘the virtual task’ section is hard to follow, the second paragraph seems a bit out of place. Please switch between the 2 ^nd^ and 3 ^rd^ paragraphs for clarity.
  a few clarification questions:
  -  Did the 3 ^rd^ object actually appear in each trial? Or only a circle marking its position?
  - What is the ‘home screen’? why should participants want to change scenes or go back to the home screen? They can skip trials?
  - Not clear if participants are allowed the full environment with the 5 objects before the experiment begins.
- Missing a description of the ‘auditory verbal’ task, along with an explanation of why this was chosen as control. Or does it refer to the RAVLT task? If so, please unify the terminology and call it RAVLT throughout the text.
- In Figure 2 appears the term ‘scrambled images’ but is not explained in the text.
- Also in figure 2 – not clear why the main session ranges from 10-45 minutes.
- In general – please unify terminology. Is “VR spatial task condition” equal to VSCT? It will help avoid confusion if it is named the same throughout the text.
- The term ‘training’ is a bit misleading, as, to my understanding, participants are not trained in performing the task itself. It should be named ‘encoding/exploration phase’.
- “when the participant accurately recalls the position of the objects after three consecutive correct trials” – this part is not clear, especially that participants can skip trials/see the correct response if they answered incorrectly. The whole outline of how a session/trial takes place from start to finish should be explained more clearly. The current division between setup and procedure makes it less clear. Please provide a united paragraph that details what the participants experiences in each trial from start to finish.
- Relatedly, if will help to reorganized figure 1 to show the sequence of events in the order participants experience them. I would even suggest to add titles on top of each figure such as “encoding phase”, “scene test trial’, “object recall task”…

Plan of analysis:

- “This will help us understand the influence of general intelligence and working memory capacity” - Which of the tests measures general intelligence? They all seem to have a memory component.

General comment about the design:

- How do the researchers plan to address the issue of interindividual variability? Comparing performance between two independent groups entails the risk that the individual abilities of participants in each group will differ a-priori. In such tasks that rely on fluid intelligence and specific cognitive abilities these differences might be more pronounced. Why not run the two tasks on the same participants? It might also require less participants overall. It seems like the design was meant for and interventional study more than for a validation study (pre-post design, two groups with alternative treatment). Either explain that this is a future aim or consider modifying the design to fit a validation study.
- I would also suggest to document ‘years of education’ for the participants, as those were shown to correlate with cognitive tasks in some cases.

Is the study design appropriate for the research question?

Partly

Is the rationale for, and objectives of, the study clearly described?

Partly

Are sufficient details of the methods provided to allow replication by others?

Partly

Are the datasets clearly presented in a useable and accessible format?

Not applicable

Reviewer Expertise:

VR, Neuropsychological testing. fMRI, eye tracking

I confirm that I have read this submission and believe that I have an appropriate level of expertise to confirm that it is of an acceptable scientific standard, however I have significant reservations, as outlined above.

# Reviewer report: Reviewer response for version 2

**DOI:** 10.21956/openreseurope.21974.r59697

**Published:** 2025-09-17

**Authors:** Jozsef Katona

**Affiliations:** 1 University of Dunaujvaros, Dunaujvaros, Hungary; 2 Óbuda University Kálmán Kandó Faculty of Electrical Engineering (Ringgold ID: 448998), Budapest, Budapest, Hungary

The manuscript presents a pre-protocol for the validation of the Virtual Spatial Configuration Task (VSCT), a novel virtual reality–based assessment tool aimed at measuring cognitive map formation abilities. Overall, the study addresses a timely and relevant gap in the field of spatial cognition research by attempting to move beyond traditional 2D assessments toward more ecologically valid immersive environments. The rationale for the study is clearly described, and the introduction provides a thorough theoretical background, situating the VSCT within the broader literature on spatial cognition, proprioceptive feedback, vestibular inputs, and the distinction between allocentric and egocentric navigation strategies. The authors convincingly argue that current methods fail to capture the multisensory, embodied nature of spatial learning, and they highlight how VR technology offers a compelling solution. This conceptual contribution is valuable and underscores the potential significance of the work.

 Despite these strengths, there are several issues that warrant further clarification and improvement. The study design, while innovative, raises some concerns. The decision to compare the VSCT with a non-spatial auditory verbal task rather than directly with the conventional Spatial Configuration Task (SCT) may limit the interpretability of the results. Although the manuscript justifies the choice by pointing to discriminant validity, omitting a direct within- or between-subject comparison between SCT and VSCT weakens the validation effort. Without such a comparison, it will be difficult to determine whether VR truly offers added value or merely represents a different modality of task presentation. A stronger design might involve a factorial approach in which SCT and VSCT are administered across conditions, allowing both convergent and discriminant validity to be assessed more directly.

 The sample size justification is commendable in its detail, particularly the use of a priori power analysis with conservative effect size assumptions. Nevertheless, the between-subjects design may be underpowered relative to the ambitious set of analyses proposed, especially given the variability inherent in VR-based testing. A discussion of how the authors plan to address potential attrition or incomplete data would strengthen the methodological section. Moreover, while demographic controls such as age and sex are mentioned, other individual differences known to affect VR performance—such as susceptibility to simulator sickness or prior gaming/VR experience—are not addressed in sufficient detail. Including such covariates could improve the robustness of the analyses.

 The description of the virtual environment is rich and detailed, and the authors’ decision to use ecologically valid household objects rather than abstract geometric figures is well motivated. This choice enhances ecological validity and participant engagement. However, it remains an open question whether the simplicity of the environments and the restriction of movement to rotational exploration are sufficient to induce the kind of cognitive map formation observed in more naturalistic navigation scenarios. While the authors argue that limiting translational movement reduces motion sickness and enhances accessibility for participants with mobility impairments, there is a trade-off in terms of ecological realism. It would be important to discuss whether this compromise could limit the generalizability of findings, particularly in applications beyond clinical or mobility-impaired populations.

 The planned statistical analyses, which include correlation matrices, t-tests, and reliability estimates, appear methodologically sound, yet they may not fully capture the complexity of the hypotheses. A simple independent-samples t-test may be insufficient to examine the interactions among group, task, and performance metrics. More advanced statistical models, such as mixed-design ANOVAs or multilevel modeling, might be better suited to handle the nested and repeated nature of the data. This would also allow the authors to assess whether learning curves or trial-by-trial performance trajectories differ systematically between VR and control groups, which would provide richer insights into the cognitive processes engaged by the VSCT.

 From a theoretical standpoint, the manuscript could benefit from a deeper discussion of how allocentric and egocentric strategies will be evaluated within the task. While the background section thoroughly reviews these concepts, the experimental design does not include a clear operationalization or manipulation of perspective-taking strategies. If the VSCT aims to advance understanding of cognitive map formation, it would be crucial to specify how the protocol disentangles the contributions of allocentric versus egocentric navigation. Similarly, the issue of immersiveness, which is central to VR research, is not systematically addressed. Established frameworks for measuring presence, such as Slater’s concepts of place illusion and plausibility, could be incorporated both as self-report measures and as explanatory variables in the analyses, thereby enriching the interpretation of the results.

 The potential applications of the VSCT are well described, particularly in the domains of neurorehabilitation and training for individuals with impaired mobility. The discussion convincingly positions the task as both an assessment tool and a potential intervention platform. However, the translational implications could be expanded further by considering broader populations, such as healthy older adults at risk of spatial disorientation, or educational contexts where spatial ability is linked to STEM achievement. Integrating these perspectives would highlight the broader societal relevance of the project.

 In terms of presentation, the manuscript is generally well written and clearly structured, with a commendable commitment to open science practices, including preregistration and data-sharing plans. Minor issues of clarity remain, particularly in distinguishing the objectives of the first and second administrations of the SCT, and in explaining how trial-by-trial feedback in the VR environment might affect learning processes relative to baseline SCT performance. These clarifications would help readers understand how the authors plan to separate task-specific effects from practice or training effects.

 In conclusion, the proposed validation of the VSCT represents a promising and original contribution to spatial cognition research. The strengths of the work lie in its innovative use of VR technology, its attention to ecological validity, and its potential clinical applications. At the same time, the study would benefit from a more rigorous validation design that directly compares the VSCT with established measures, a more sophisticated analytic strategy, and an explicit operationalization of allocentric and egocentric strategies. Addressing these issues would significantly enhance the scientific impact of the work and provide a more compelling case for the VSCT as a valid and useful tool in both research and applied settings.

Is the study design appropriate for the research question?

Partly

Is the rationale for, and objectives of, the study clearly described?

Partly

Are sufficient details of the methods provided to allow replication by others?

Yes

Are the datasets clearly presented in a useable and accessible format?

Not applicable

Reviewer Expertise:

Human-computer interaction; cognitive psychology; software engineering.

I confirm that I have read this submission and believe that I have an appropriate level of expertise to confirm that it is of an acceptable scientific standard, however I have significant reservations, as outlined above.

# Response

**Published:** 2025-09-30

**Authors:** Giorgio Li Pira

**Affiliations:** Psicologia, Universita degli Studi di Bologna Dipartimento di Psicologia Renzo Canestrari, Bologna, Emilia-Romagna, Italy

The manuscript presents a pre-protocol for the validation of the Virtual Spatial Configuration Task (VSCT), a novel virtual reality–based assessment tool aimed at measuring cognitive map formation abilities. Overall, the study addresses a timely and relevant gap in the field of spatial cognition research by attempting to move beyond traditional 2D assessments toward more ecologically valid immersive environments. The rationale for the study is clearly described, and the introduction provides a thorough theoretical background, situating the VSCT within the broader literature on spatial cognition, proprioceptive feedback, vestibular inputs, and the distinction between allocentric and egocentric navigation strategies. The authors convincingly argue that current methods fail to capture the multisensory, embodied nature of spatial learning, and they highlight how VR technology offers a compelling solution. This conceptual contribution is valuable and underscores the potential significance of the work.

 Despite these strengths, there are several issues that warrant further clarification and improvement. The study design, while innovative, raises some concerns. The decision to compare the VSCT with a non-spatial auditory verbal task rather than directly with the conventional Spatial Configuration Task (SCT) may limit the interpretability of the results. Although the manuscript justifies the choice by pointing to discriminant validity, omitting a direct within- or between-subject comparison between SCT and VSCT weakens the validation effort. Without such a comparison, it will be difficult to determine whether VR truly offers added value or merely represents a different modality of task presentation. A stronger design might involve a factorial approach in which SCT and VSCT are administered across conditions, allowing both convergent and discriminant validity to be assessed more directly.

*We thank the reviewer for this important observation. At this stage of our research, our primary aim is to validate the effectiveness of the VSCT as an assessment tool for spatial abilities, using the SCT as the benchmark (as specified in the text). We fully agree that a factorial design directly comparing SCT and VSCT across conditions would provide a stronger framework to assess the experimental task, as well as the potential added value of VR. However, a systematic investigation of the benefits of virtual reality in training spatial abilities goes beyond the scope of the present study. Such an evaluation is planned as a next step in our research program, once the validity of the VSCT assessment has been firmly established.*

 The sample size justification is commendable in its detail, particularly the use of a priori power

 analysis with conservative effect size assumptions. Nevertheless, the between-subjects design may be underpowered relative to the ambitious set of analyses proposed, especially given the variability inherent in VR-based testing. A discussion of how the authors plan to address potential attrition or incomplete data would strengthen the methodological section. Moreover, while demographic controls such as age and sex are mentioned, other individual differences known to affect VR performance—such as susceptibility to simulator sickness or prior gaming/VR experience—are not addressed in sufficient detail. Including such covariates could improve the robustness of the analyses.  

*Thank you for this valuable feedback. While we recognize the concern that the proposed between-subjects design may be underpowered for the full range of planned analyses, we respectfully believe that our target sample size of **N = 166** is sufficient. This estimate is based on a conservative a priori power analysis, which accounts for the variability typically observed in VR-based tasks.* *Moreover, we have revised the manuscript to address these concerns. Specifically, we added the following statement to the methodological section: “Attrition and incomplete data will be addressed through oversampling to compensate for potential dropouts, and by implementing standardized training and familiarization procedures to reduce task-related discontinuation. Participants who withdraw or produce incomplete data will be documented, and their exclusion will be transparently reported.”* *In addition, we would like to highlight that we included the Simulator Sickness Questionnaire (SSQ) as a measure to assess individual differences in susceptibility to simulator sickness, thereby allowing us to account for this factor in our analyses.*  

 The description of the virtual environment is rich and detailed, and the authors’ decision to use ecologically valid household objects rather than abstract geometric figures is well motivated. This choice enhances ecological validity and participant engagement. However, it remains an open question whether the simplicity of the environments and the restriction of movement to rotational exploration are sufficient to induce the kind of cognitive map formation observed in more naturalistic navigation scenarios. While the authors argue that limiting translational movement reduces motion sickness and enhances accessibility for participants with mobility impairments, there is a trade-off in terms of ecological realism. It would be important to discuss whether this compromise could limit the generalizability of findings, particularly in applications beyond clinical or mobility-impaired populations.  

*We acknowledge the trade-off inherent in our design and are aware of the potential limitations in statistical power. However, we consider it safe to assume that cognitive map formation is reliably triggered in our paradigm, as the task is based on the Spatial Configuration Task (SCT; Ford et al., 2017), which has already been shown to assess this ability effectively with no movement and only simple geometrical shapes.*

 The planned statistical analyses, which include correlation matrices, t-tests, and reliability estimates, appear methodologically sound, yet they may not fully capture the complexity of the hypotheses. A simple independent-samples t-test may be insufficient to examine the interactions among group, task, and performance metrics. More advanced statistical models, such as mixed-design ANOVAs or multilevel modeling, might be better suited to handle the nested and repeated nature of the data. This would also allow the authors to assess whether learning curves or trial-by-trial performance trajectories differ systematically between VR and control groups, which would provide richer insights into the cognitive processes engaged by the VSCT.

*We agree that more advanced statistical models could be highly informative for exploring learning curves and trial-by-trial trajectories. However, at this stage, our primary focus is not on evaluating between-group performance, but rather on validating the VSCT as a tool. On this basis, we have planned for future work to compare the VSCT with an auditory verbal task, which as of now serve as a divergent validity measure.* *We have now modified the manuscript accordingly:*

1. ***Clarification on paragraph heading*** *: We changed the heading of the paragraph that may have generated confusion (from “Correlation analysis and t-test” to “Correlation analysis”) to better reflect its content and avoid misinterpretation.*
2. ***Statistical analysis*** *: We clarify that our intention is not to analyze pre- vs post-training changes, but rather to ensure that the two groups do not differ significantly on relevant baseline scores. For this purpose, independent-samples t-tests are sufficient and appropriate.*

From a theoretical standpoint, the manuscript could benefit from a deeper discussion of how allocentric and egocentric strategies will be evaluated within the task. While the background section thoroughly reviews these concepts, the experimental design does not include a clear operationalization or manipulation of perspective-taking strategies. If the VSCT aims to advance understanding of cognitive map formation, it would be crucial to specify how the protocol disentangles the contributions of allocentric versus egocentric navigation. Similarly, the issue of immersiveness, which is central to VR research, is not systematically addressed. Established frameworks for measuring presence, such as Slater’s concepts of place illusion and plausibility, could be incorporated both as self-report measures and as explanatory variables in the analyses, thereby enriching the interpretation of the results.  

*We appreciate the suggestion to include an evaluation of the immersiveness of the VR environment. However, the main aim of our study is not to compare levels of immersiveness, but rather to investigate 2D vs. 3D approaches to the assessment of spatial memory, as highlighted in the manuscript. In particular, VR allows not only the evaluation of 3D maps in a fully immersive environment, but also the incorporation of sensorimotor feedback related to body movement, which provides an additional source of input for cognitive map formation and enables a more ecological and realistic assessment* *" VR offers several advantages over traditional 2D and desktop 3D assessments of spatial abilities, including the ability to evaluate 3D maps in a fully immersive 3D environment. Additionally, VR can incorporate sensory information related to body movement, providing an additional source of input for cognitive map formation, thereby enabling a more ecological and realistic assessment. Therefore, we propose a protocol for validating a novel VR task, the Virtual Spatial Cognition Task (VSCT). This task simulates participants' cognitive map formation abilities by requiring them to explore a virtual environment, learn spatial relationships between landmarks, and recall their positions."* *Given this theoretical rationale and the current experimental design, we believe that adding a questionnaire on immersiveness would not be essential, as we do not formulate specific hypotheses regarding immersivity and it is not the primary focus of the study. Nevertheless, after the evaluation of the present study, we agree that incorporating an assessment of immersiveness could be a valuable addition for future investigations.*

 The potential applications of the VSCT are well described, particularly in the domains of neurorehabilitation and training for individuals with impaired mobility. The discussion convincingly positions the task as both an assessment tool and a potential intervention platform. However, the translational implications could be expanded further by considering broader populations, such as healthy older adults at risk of spatial disorientation, or educational contexts where spatial ability is linked to STEM achievement. Integrating these perspectives would highlight the broader societal relevance of the project.

*We appreciate this valuable suggestion. As recommended, we expanded the discussion section to consider broader populations beyond clinical groups. “Beyond clinical populations, the VSCT could also hold significant value for broader groups at risk of spatial difficulties. For example, healthy older adults often experience age-related declines in spatial orientation that increase the risk of disorientation and falls; the VSCT may provide an engaging and safe way to monitor and train these abilities proactively. Similarly, in educational contexts, spatial ability is closely linked to achievement in STEM disciplines. Incorporating the VSCT into training curricula could provide students with opportunities to strengthen cognitive map formation skills that underlie problem-solving in fields such as mathematics, engineering, and the natural sciences. By extending its scope to these populations, the VSCT not only serves as a rehabilitative and diagnostic tool but also as a platform with the potential to foster resilience, independence, and academic success”. Specifically, we now highlight the potential applications of the VSCT for healthy older adults at risk of spatial disorientation, as well as for educational contexts where spatial ability is linked to STEM achievement. This addition emphasizes the broader societal relevance of the project and positions the VSCT as both a rehabilitative/diagnostic tool and a platform with potential benefits for resilience, independence, and academic success.*

 In terms of presentation, the manuscript is generally well written and clearly structured, with a commendable commitment to open science practices, including preregistration and data-sharing plans. Minor issues of clarity remain, particularly in distinguishing the objectives of the first and second administrations of the SCT, and in explaining how trial-by-trial feedback in the VR environment might affect learning processes relative to baseline SCT performance. These clarifications would help readers understand how the authors plan to separate task-specific effects from practice or training effects.

 In conclusion, the proposed validation of the VSCT represents a promising and original contribution to spatial cognition research. The strengths of the work lie in its innovative use of VR technology, its attention to ecological validity, and its potential clinical applications. At the same time, the study would benefit from a more rigorous validation design that directly compares the VSCT with established measures, a more sophisticated analytic strategy, and an explicit operationalization of allocentric and egocentric strategies. Addressing these issues would significantly enhance the scientific impact of the work and provide a more compelling case for the VSCT as a valid and useful tool in both research and applied settings.

# Reviewer report: Reviewer response for version 2

**DOI:** 10.21956/openreseurope.21974.r55020

**Published:** 2025-09-03

**Authors:** Marta Matamala-Gomez

**Affiliations:** 1 University of Barcelona, Barcelona, Spain

The present study aims to compare the conventional Spatial Configuration task (SCT) with a Virtual SCT conducted in a virtual reality environment. However, in the present study, the authors decided to conduct a between-subjects study comparing the virtual SCT with a non-spatial auditory verbal task. 

 In my opinion, since this task in virtual reality has not yet been validated, the authors should focus on comparing the conventional Spatial Configuration Task with the one conducted in virtual reality. In this way, the authors can demonstrate why or what the benefits of using VR systems are when conducting the SCT.

 Figure 1. shows a pictorial representation of the supposed VR environment to be used. I think that the authors would benefit from representing more realistic (home-based) VR environments like a living room, a kitchen... in which the participants can select some objects that are used to be placed in such environments.   

 The authors talk about differences when conducting the SCT from an egocentric or allocentric view (where they are placed in relation to the objects). However, they do not specify any comparison or test between the two perspectives in their experimental design. 

 I would like to suggest the following modification in their experimental timeline: I would compare VR SCTask vs. Conventional SCTask. Then, I will compare the ego vs. allocentric point of view in the two groups. 

 The experimental design I propose is a 2x2 between-subjects design, with two main groups: VR SCT vs. Non-VR SCT, and 2 conditions in each group: Egocentric vs. Allocentric view. 

 Did the authors think of adding some evaluation of the immersiveness of the VR environment and how this can influence or correlate with the results when conducting the SCTask? Here, I give you some references to check how to evaluate it: Slater, M. (2009). Place illusion and plausibility can lead to realistic behaviour in immersive virtual environments. *Philosophical Transactions of the Royal Society B: Biological Sciences*, *364*(1535), 3549-3557.

 The authors should add more relevant bibliography using immersive VR to improve spatial memory and cognitive maps. Here, I give you some references:

 Refer 1 to 3

 Also, there are other studies in VR showing differences between the allocentric and egocentric points of view in VR: 

 Refer 4 to 7

 In the results section, the authors should show if there is an impact of being immersed in aVR environment when conducting the SCTask, and which is the impact of egocentric or allocentric perspectives is when conducting the SCT on spatial memory. 

 Finally, the authors should discuss which is the added value of using VR systems to improve spatial memory compared to conventional techniques. 

 In the analysis section, the authors can use an ANOVA two-factor or other statistical test, more complete than a t-test, which is a very simple test mainly used to assess pre-post training changes. 

Is the study design appropriate for the research question?

Partly

Is the rationale for, and objectives of, the study clearly described?

Partly

Are sufficient details of the methods provided to allow replication by others?

Yes

Are the datasets clearly presented in a useable and accessible format?

Not applicable

Reviewer Expertise:

Cognitive neuroscience, virtual reality, virtual embodiment, motor learning, pain perception

I confirm that I have read this submission and believe that I have an appropriate level of expertise to state that I do not consider it to be of an acceptable scientific standard, for reasons outlined above.

## References

[1] : Neurorehabilitation of Spatial Memory Using Virtual Environments: A Systematic Review. *Journal of Clinical Medicine* .2019;8(10) : 10.3390/jcm8101516 10.3390/jcm8101516 PMC683310931547137

[2] : How different spatial representations interact in virtual environments: the role of mental frame syncing. *Cognitive Processing* .2015;16(2) : 10.1007/s10339-015-0646-4 191-201 10.1007/s10339-015-0646-4 25663561

[3] : Disentangling the Contribution of Spatial Reference Frames to Executive Functioning in Healthy and Pathological Aging: An Experimental Study with Virtual Reality. *Sensors* .2018;18(6) : 10.3390/s18061783 10.3390/s18061783 PMC602211629865196

[4] : Egocentric and Allocentric Spatial Memory for Body Parts: A Virtual Reality Study. *Journal of Cognition* .2024;7(1) : 10.5334/joc.357 10.5334/joc.357 38638460 PMC11025578

[5] : Detecting early egocentric and allocentric impairments deficits in Alzheimerâ€™s disease: an experimental study with virtual reality. *Frontiers in Aging Neuroscience* .2015;7: 10.3389/fnagi.2015.00088 10.3389/fnagi.2015.00088 26042034 PMC4438252

[6] : The effect of visual perspective on episodic memory in aging: A virtual reality study. *Consciousness and Cognition* .2023;116: 10.1016/j.concog.2023.103603 10.1016/j.concog.2023.103603 37976783

[7] : Do not get lost in translation: The role of egocentric heading in spatial orientation. *Neuroscience Letters* .2015;602: 10.1016/j.neulet.2015.06.057 84-88 10.1016/j.neulet.2015.06.057 26145315

# Response

**Published:** 2025-09-30

**Authors:** Giorgio Li Pira

**Affiliations:** Psicologia, Universita degli Studi di Bologna Dipartimento di Psicologia Renzo Canestrari, Bologna, Emilia-Romagna, Italy

The present study aims to compare the conventional Spatial Configuration task (SCT) with a Virtual SCT conducted in a virtual reality environment. However, in the present study, the authors decided to conduct a between-subjects study comparing the virtual SCT with a non-spatial auditory verbal task. 

 In my opinion, since this task in virtual reality has not yet been validated, the authors should focus on comparing the conventional Spatial Configuration Task with the one conducted in virtual reality. In this way, the authors can demonstrate why or what the benefits of using VR systems are when conducting the SCT.

*Thank you for your comment. As we explain in the text, “We will run correlations matrix between CORSI, SBSOD, and hits at both the VSCT and SCT. This will help us understand the influence of general intelligence and working memory capacity on spatial ability and ensure that the VSCT and SCT are comparable tasks.” Therefore, we have indeed planned a comparison between the two tasks to ensure their comparability and to assess the specific benefits of implementing the SCT in virtual reality.*

 Figure 1. shows a pictorial representation of the supposed VR environment to be used. I think that the authors would benefit from representing more realistic (home-based) VR environments like a living room, a kitchen... in which the participants can select some objects that are used to be placed in such environments.   

*While we agree that a more realistic home-based VR environment (e.g., living room, kitchen) could be visually engaging, such settings would introduce too many spatial reference points. This would reduce the task’s ability to isolate spatial memory and configuration skills. For this reason, we decided to keep only the objects themselves in the environment and not include any additional points of reference.*

 The authors talk about differences when conducting the SCT from an egocentric or allocentric view (where they are placed in relation to the objects). However, they do not specify any comparison or test between the two perspectives in their experimental design. 

*Thank you for this observation. In our design, as in real life, egocentric and allocentric strategies are always intermixed. The task is presented in first person and thus provides only an **egocentric view** (to avoid confusion, the bird’s-eye view shown in the figure, is included only to illustrate the layout of the environment). From this egocentric perspective, participants must extrapolate an **allocentric cognitive map** in order to efficiently complete the task. Our aim is not to directly compare egocentric and allocentric strategies, but rather, as highlighted in the text, to compare the VSCT with the SCT.*

 I would like to suggest the following modification in their experimental timeline: I would compare VR SCTask vs. Conventional SCTask. Then, I will compare the ego vs. allocentric point of view in the two groups. 

 The experimental design I propose is a 2x2 between-subjects design, with two main groups: VR SCT vs. Non-VR SCT, and 2 conditions in each group: Egocentric vs. Allocentric view. 

*We carefully considered your suggestions and discussed them thoroughly among the author team. While we greatly value your input, we believe that some of your proposed modifications are not fully applicable to the specific aims and design of the present protocol.* *As you may have seen from the previous review rounds, the manuscript has already undergone substantial revision and clarification, and data collection has now begun following the initial feedback received. At this stage, extensive structural changes would not be feasible without altering the scope and preregistered methodology of the study.* *In particular, regarding your request to include egocentric versus allocentric conditions: we fear there may have been a misunderstanding. The distinction between egocentric and allocentric strategies is already discussed in the theoretical background (see Strategies of spatial orientation and navigation paragraph). However, in our design it is treated as a theoretical framework guiding the rationale, not as an experimental factor to be manipulated. Introducing it as an independent variable at this stage would shift the study away from its primary objective—namely, validating the VSCT through convergent and discriminant validity analyses. We agree that systematically testing egocentric versus allocentric perspectives would be an excellent direction for future experimental work, especially in the context of training, but it goes beyond the present validation study.* *We hope this clarifies our methodological choices and we remain very grateful for your constructive engagement with our work.*

 Did the authors think of adding some evaluation of the immersiveness of the VR environment and how this can influence or correlate with the results when conducting the SCTask? Here, I give you some references to check how to evaluate it: Slater, M. (2009). Place illusion and plausibility can lead to realistic behaviour in immersive virtual environments. 

*Philosophical Transactions of the Royal Society B: Biological Sciences*, *364*(1535), 3549-3557.

*We appreciate the suggestion to include an evaluation of the immersiveness of the VR environment and acknowledge the relevance of work such as Slater (2009). However, the main aim of our study is not to compare levels of immersiveness, but rather to investigate **2D vs. 3D approaches to the assessment of spatial memory**, as highlighted in the manuscript. In particular, VR allows not only the evaluation of 3D maps in a fully immersive environment, but also the incorporation of **sensorimotor feedback related to body movement**, which provides an additional source of input for cognitive map formation and enables a more ecological and realistic assessment* *" VR offers several advantages over traditional 2D and desktop 3D assessments of spatial abilities, including the ability to evaluate 3D maps in a fully immersive 3D environment. Additionally, VR can incorporate sensory information related to body movement, providing an additional source of input for cognitive map formation, thereby enabling a more ecological and realistic assessment. Therefore, we propose a protocol for validating a novel VR task, the Virtual Spatial Cognition Task (VSCT). This task simulates participants' cognitive map formation abilities by requiring them to explore a virtual environment, learn spatial relationships between landmarks, and recall their positions."* *Given this theoretical rationale and the current experimental design, we believe that adding a questionnaire on immersiveness would not be essential, as we do not formulate specific hypotheses regarding immersivity and it is not the primary focus of the study. Nevertheless, after the evaluation of the present study, we agree that incorporating an assessment of immersiveness could be a valuable addition for future investigations.*

 The authors should add more relevant bibliography using immersive VR to improve spatial memory and cognitive maps. Here, I give you some references:

 Refer 1 to 3

 Also, there are other studies in VR showing differences between the allocentric and egocentric points of view in VR: 

 Refer 4 to 7

 In the results section, the authors should show if there is an impact of being immersed in aVR environment when conducting the SCTask, and which is the impact of egocentric or allocentric perspectives is when conducting the SCT on spatial memory. 

 Finally, the authors should discuss which is the added value of using VR systems to improve spatial memory compared to conventional techniques. 

 In the analysis section, the authors can use an ANOVA two-factor or other statistical test, more complete than a t-test, which is a very simple test mainly used to assess pre-post training changes. *We have now modified the manuscript accordingly:*

1. ***Clarification on paragraph heading*** *: We changed the heading of the paragraph that could have generated some confusion (from “ **Correlation analysis and t-test**” to “ **Correlation analysis**”), in order to better reflect its content and avoid misinterpretation.*
2. ***Statistical analysis*** *: We would like to clarify that our intention is not to analyze pre- vs post-training changes, but rather to ensure that the two groups do not differ significantly in the relevant baseline scores. For this purpose, we plan to use independent t-tests, which are appropriate for this specific aim.*

# Reviewer report: Reviewer response for version 2

**DOI:** 10.21956/openreseurope.21974.r54387

**Published:** 2025-06-09

**Authors:** Simon Lhuillier

**Affiliations:** 1 Université Gustave Eiffel, Université de Paris, Versailles, France

I would like to thank the authors for their willingness to respond to the reviewers as best they could and for the modifications they made to their manuscript as a result. Although the methodological choices made for this article still seem questionable to me, I also admit that this evaluation may stem from a difference of field or practice. Taking this into account, as well as the opinions of my colleagues and the scope of the article as defended by the authors, I am willing to align myself with a relatively favourable opinion.

Is the study design appropriate for the research question?

No

Is the rationale for, and objectives of, the study clearly described?

Partly

Are sufficient details of the methods provided to allow replication by others?

No

Are the datasets clearly presented in a useable and accessible format?

Not applicable

Reviewer Expertise:

Spatial cognition, spatial representations, virtual reality, spatial and temporal cognitive interactions, embodied and situated cognition

I confirm that I have read this submission and believe that I have an appropriate level of expertise to confirm that it is of an acceptable scientific standard, however I have significant reservations, as outlined above.

# Reviewer report: Reviewer response for version 1

**DOI:** 10.21956/openreseurope.21031.r51677

**Published:** 2025-03-21

**Authors:** Alastair D. Smith

**Affiliations:** 1 University of Plymouth, Plymouth, UK

The study protocol outlines a plan for validating an immersive virtual reality variant of the Spatial Configuration Task (SCT), a behavioural measure designed to provide an insight into the construction of cognitive maps. The authors plan to conduct sessions based upon administration of the original SCT, to be followed by the VR equivalent (VSCT), and then a repeat administration of the SCT. Participants will also complete the Santa Barbara Sense of Direction Scale, the Rey Auditory Verbal Learning Test, and backward Corsi blocks, and the key predictions are that 1) SCT performance will correlate with VSCT performance (thus validating the immersive variant), and 2) that both SCT tasks will correlate with Corsi performance (supporting a proposed relationship between working memory and cognitive map construction).

 The broad ideas here are interesting, and the aim to validate a task in VR makes an awful lot of sense. I am particularly sensitive to discussion of the role that idiothetic information might play in the large-scale immersive version, as this is an important dialogue in the current literature. That said, I do feel that the protocol raises more questions than it perhaps answers. I shall summarise my key thoughts here, in no particular order:

 1. The methodological detail is somewhat sparse for the key tasks, the SCT being particularly difficult to follow.

 2. Similarly, the predictions are rather Spartan, and seem to focus solely on a small number of intercorrelations. The tasks additional to SCT, VSCT, and Corsi are not mentioned at all in the ‘expected results’ section which, in the first instance, leads one to wonder why they have been included. What exactly does the SCT tell us about cognitive maps, and how they might differ between individuals? Granularity would really help here.

 3. The relationship between the first and final administration of the original SCT task is unclear. The concept of training and improvement is hinted at in the Discussion section, and one wonders if repeated administration of the task is somehow seen to be an intervention. If so, a more formal delineation of this idea, and the evidence supporting it, needs to be presented. Moreover, what role is the VSCT task supposed to play here – i.e. is it expected to somehow catalyse that training effect?

 4. I’m not particularly convinced by the argument that rotational movements necessarily meet the exploratory claims made by the authors whilst also making the task accessible to groups with mobility difficulties. That is, why could participants not physically explore the space with full body movements? If it is essential that exploration can only be rotational in the SCT, then the argument for it being useful for participants with mobility issues is moot. If it is not essential, then why not start with something that allows for more natural exploratory movements and then consider validating a more accessible rotational version? In short, it is unclear exactly what is motivating the design as presented here.  

 5. In order to be convinced that the SCT measures cognitive map construction, and that the VSCT does so in a similar manner, might it not also be useful to run an additional task that has a stronger evidence base for tapping the construct in question (e.g. four mountains, place learning, etc. – notwithstanding theoretical arguments about what constitutes a cognitive map, or whether those measures also test it!)

 6. Finally, there appears to be no sense of addressing what the VSCT might buy us. If performance relates to a desktop equivalent, then what will be the need to continue developing a VR version? One might hope that performance will be superior, in the VR version, or more strongly predicted by related factors, thus supporting its potential as a more valid and accurate assay.

Is the study design appropriate for the research question?

Partly

Is the rationale for, and objectives of, the study clearly described?

Partly

Are sufficient details of the methods provided to allow replication by others?

No

Are the datasets clearly presented in a useable and accessible format?

Not applicable

Reviewer Expertise:

Spatial cognition; Neuropsychology; Development; Ageing; Virtual Reality

I confirm that I have read this submission and believe that I have an appropriate level of expertise to confirm that it is of an acceptable scientific standard, however I have significant reservations, as outlined above.

# Response

**Published:** 2025-04-26

**Authors:** Giorgio Li Pira

**Affiliations:** Psicologia, Universita degli Studi di Bologna Dipartimento di Psicologia Renzo Canestrari, Bologna, Emilia-Romagna, Italy

1. The methodological detail is somewhat sparse for the key tasks, the SCT being particularly difficult to follow.   Thank you for your comment. While we acknowledge that the description of the Spatial Configuration Task (SCT) is brief, our primary focus in this manuscript is the development and validation of the Virtual Spatial Configuration Task (VSCT). The SCT has been thoroughly described and validated in the referenced publication (Burles et al., 2017), which we cite for readers seeking detailed methodological information. Given the scope of this study, we chose not to include redundant details and instead directed our attention to the novel aspects of the VSCT. 

 2. Similarly, the predictions are rather Spartan, and seem to focus solely on a small number of intercorrelations. The tasks additional to SCT, VSCT, and Corsi are not mentioned at all in the ‘expected results’ section which, in the first instance, leads one to wonder why they have been included. What exactly does the SCT tell us about cognitive maps, and how they might differ between individuals? Granularity would really help here.  We recognize that the original Expected Results section was concise and primarily centered on predicted intercorrelations. However, we would like to clarify that our central aim is the validation of the VSCT as a measure of cognitive map formation, which is why our predictions were primarily focused on its relationship with SCT and Corsi—both of which have been previously linked to spatial memory and working memory processes involved in cognitive mapping.  The inclusion of additional tasks such as the RAVLT and the SSQ serves specific, complementary purposes: RAVLT acts as a control to distinguish domain-specific (spatial) from domain-general (verbal) memory processes, and the SSQ assesses the feasibility and user comfort of the VR environment. While we did not explicitly state the expected outcomes for these tasks in the results section, we have updated the manuscript to clarify their role and the rationale behind their inclusion.  As for the SCT, we rely on existing literature (e.g., Burles et al., 2017) which demonstrates its utility in assessing allocentric spatial representations. We will clarify how SCT performance reflects individual differences in forming and retrieving cognitive maps and how it serves as a benchmark for validating the VSCT. 

 3. The relationship between the first and final administration of the original SCT task is unclear. The concept of training and improvement is hinted at in the Discussion section, and one wonders if repeated administration of the task is somehow seen to be an intervention. If so, a more formal delineation of this idea, and the evidence supporting it, needs to be presented. Moreover, what role is the VSCT task supposed to play here – i.e. is it expected to somehow catalyse that training effect?   At this stage, our primary objective is to validate the VSCT as an effective and sensitive measure of cognitive map formation ability.  While the repeated administration of the SCT might hint at potential training effects, our current study is not designed to assess those. Instead, it is structured to determine whether the VSCT can reliably detect individual differences in spatial performance and whether it correlates with established spatial measures like the SCT. Establishing the task’s validity is a necessary step before we can confidently use it to assess intervention outcomes or training effects. Once validated, future studies will be able to explore its utility in those contexts.  To this end, only the first administration of the SCT (T1) will be used for correlation analyses with the VSCT, as it provides a baseline measure of spatial performance. The second administration (T2) is included solely for exploratory purposes—to investigate whether repeated exposure might lead to performance improvements and to preliminarily explore potential training effects. These exploratory findings could inform the design of future studies specifically aimed at assessing the impact of training or interventions. Establishing the VSCT’s validity is a necessary first step before we can confidently use it in such longitudinal or interventional contexts. 

 4. I’m not particularly convinced by the argument that rotational movements necessarily meet the exploratory claims made by the authors whilst also making the task accessible to groups with mobility difficulties. That is, why could participants not physically explore the space with full body movements? If it is essential that exploration can only be rotational in the SCT, then the argument for it being useful for participants with mobility issues is moot. If it is not essential, then why not start with something that allows for more natural exploratory movements and then consider validating a more accessible rotational version? In short, it is unclear exactly what is motivating the design as presented here.    We appreciate the opportunity to clarify our design choices. The decision to restrict participant movement to rotational exploration, rather than allowing full-body translational navigation, was intentionally made to enhance accessibility and reduce complexity for individuals with mobility impairments. While we agree that physical locomotion may offer more natural exploratory movements, our primary aim was to create a tool that is not only ecologically valid but also inclusive and feasible for populations who may be unable to ambulate.  We have expanded the manuscript to better articulate this rationale. As described in the revised text, rotational exploration provides participants with vestibular and proprioceptive feedback critical to spatial learning, while avoiding the challenges and discomfort (e.g., motion sickness, cognitive load from complex controls) often associated with translational movement in VR. Moreover, this simplified approach mirrors the original SCT structure and allows us to maintain methodological continuity while still leveraging the immersive benefits of VR. 

 5. In order to be convinced that the SCT measures cognitive map construction, and that the VSCT does so in a similar manner, might it not also be useful to run an additional task that has a stronger evidence base for tapping the construct in question (e.g. four mountains, place learning, etc. – notwithstanding theoretical arguments about what constitutes a cognitive map, or whether those measures also test it!)   Thank you for this valuable suggestion. We fully acknowledge that cognitive map formation is a debated construct, with multiple theoretical models and a range of tasks—such as Four Mountains or place learning—proposed to assess it. However, as we outline in the manuscript, our interest lies not only in measuring cognitive map formation broadly, but specifically in assessing the ability to switch between egocentric and allocentric reference frames during spatial encoding and retrieval.  Given this focus, we selected the Spatial Configuration Task (SCT) as the most appropriate comparison, as it has been shown to emphasize these perspective transformations from a behavioral and neural perspective and has been used in previous studies exploring similar spatial mechanisms. This alignment in cognitive demands made the SCT a meaningful benchmark for validating the VSCT. 

 6. Finally, there appears to be no sense of addressing what the VSCT might buy us. If performance relates to a desktop equivalent, then what will be the need to continue developing a VR version? One might hope that performance will be superior, in the VR version, or more strongly predicted by related factors, thus supporting its potential as a more valid and accurate assay.  As we briefly mention in the Discussion, our long-term vision for the VSCT extends beyond assessment. While the current study is focused on validating its utility as a tool to measure cognitive map formation, we believe the real strength of the VR-based approach lies in its potential as an interactive training platform.  Unlike traditional desktop-based assessments, the VSCT incorporates proprioceptive and vestibular feedback, providing a more immersive and embodied experience. These sensory components are critical in real-world navigation and spatial learning, and their inclusion allows us to design a tool that not only measures cognitive map formation but may also be used to train and monitor improvements in spatial abilities over time—particularly for populations with motor or cognitive impairments.  Additionally, the ecological validity afforded by VR enhances our ability to study nuanced cognitive processes, such as the switching between egocentric and allocentric frames of reference. This may help us better differentiate the underlying mechanisms that support spatial orientation and map formation, something that more constrained desktop tasks may not capture as effectively.

# Reviewer report: Reviewer response for version 1

**DOI:** 10.21956/openreseurope.21031.r51458

**Published:** 2025-03-19

**Authors:** David Uttal

**Affiliations:** 1 Northwestern University, Evanston, USA

This manuscript reports on the development and validation of a new measure of spatial cognition, the Virtual Spatial Configuration Task (VSCT). The new task creates a virtual environment in which participants explore using a wheeled chair. The authors suggest that this new test offers several advantages over existing measures, including the elimination of cognitive projection from two to three dimensions and a (hypothesized) reduction in motion sickness.

 I agree that the proposed test could serve a very important role in both research and training. However, there are several important issues that must be addressed:

 a) The actual research question to be tested is unclear. What precisely will be tested? What comparison (or correlation) was the power analysis designed to address? There appear to be several possible answers to this question. Perhaps the focus is on the validation of the test, and therefore the critical question is about correlations with other measures. Alternatively, the focus could be on a comparison to of the two tasks (spatial versus verbal). I realize that both possibilities are likely true, but nevertheless, the authors need to be clear about what specifically is being pre-registered.

 Obviously, this is the most important question that must be addressed. The fact that the specific research question being addressed and pre-registered is unclear is a serious problem. The researchers need to be much clearer as to what questions they are testing, the focus of the power analysis, and so on.

 b) The theoretical motivation for some of the experimental manipulations is unclear. Specifically, it is not clear why the researchers included the experimental manipulation in which half of the participants do NOT complete the spatial test but rather a “non-spatial auditory verbal task to isolate memory from spatial reasoning.” This explanation is inadequate, particularly given the centrality of this manipulation in the experimental design. More information is needed regarding what specifically the alternate task is designed to control for and what construct it is designed to operationalize and measure.

 c) The work does not seem to adequately build on prior work with virtual reality. There are now several publicly available VR-based tests of spatial learning (e.g., Weisberg, S. M., & Newcombe, N. S. (2016)[Refer 1]). Admittedly, many of these tests rely on non-immersive VR, but that alone is not a reason to dismiss them without consideration. What specific weaknesses or limitations of prior research will the proposed test address?

 d) The researchers do not consider possible disadvantages or challenges of the “chair-based perspective.” While the researchers are correct regarding possible advantages, this method may also introduce disadvantages. For example, the vestibular cues associated with moving in a chair may be very different from those involved in walking. Can we assume that the unfamiliar chair-based perspective will provide a valid measure of navigation skill in everyday life experience? Why or why not?

 e) Some aspects of the methods are underspecified. Basic information about the test, including the number of trials, is not included. Also, how will the psychometric reliability be assessed? Is there any preliminary data to suggest the test will be reliable? Likewise, the discussion of the expected results seems underspecified. For example, the authors predict positive correlations between some of the measures, but most tests show some positive relationship with each other. Thus, simply showing a positive correlation is not enough to claim that the test has been validated. Therefore, a more specific prediction regarding what level of correlation would be considered evidence of validation is needed.

 f) One minor issue: Too many abbreviations are used, and they are used too frequently, sometimes within the same sentence. This makes it difficult to follow the text.

Is the study design appropriate for the research question?

Partly

Is the rationale for, and objectives of, the study clearly described?

Partly

Are sufficient details of the methods provided to allow replication by others?

No

Are the datasets clearly presented in a useable and accessible format?

Not applicable

Reviewer Expertise:

Spatial cognition

I confirm that I have read this submission and believe that I have an appropriate level of expertise to confirm that it is of an acceptable scientific standard, however I have significant reservations, as outlined above.

## References

[1] : How do (some) people make a cognitive map? Routes, places, and working memory. *J Exp Psychol Learn Mem Cogn* .2016;42(5) : 10.1037/xlm0000200 768-785 10.1037/xlm0000200 26595065

# Response

**Published:** 2025-04-26

**Authors:** Giorgio Li Pira

**Affiliations:** Psicologia, Universita degli Studi di Bologna Dipartimento di Psicologia Renzo Canestrari, Bologna, Emilia-Romagna, Italy

a) The actual research question to be tested is unclear. What precisely will be tested? What comparison (or correlation) was the power analysis designed to address? There appear to be several possible answers to this question. Perhaps the focus is on the validation of the test, and therefore the critical question is about correlations with other measures.

 Alternatively, the focus could be on a comparison to of the two tasks (spatial versus verbal). I realize that both possibilities are likely true, but nevertheless, the authors need to be clear about what specifically is being pre-registered.    

 Thank you for this important and constructive comment. We fully agree that the specific research question and the rationale for the power analysis must be clearly stated. We have revised the manuscript accordingly to clarify both the theoretical framework and the statistical approach. 

 The primary objective of the study is to validate the Virtual Spatial Configuration Task (VSCT) as a measure of cognitive map formation abilities. This validation involves testing:  Convergent validity, through correlations between the VSCT and two established spatial cognition measures (the Spatial Configuration Task [SCT] and the Backward Corsi Block-Tapping Task), and  Discriminant validity, by comparing the correlation between SCT and VSCT with the correlation between SCT and a verbal episodic memory task (the Rey Auditory Verbal Learning Test [RAVLT]). 

 To test discriminant validity directly, a between-subjects design is used in which one group completes the VSCT and SCT (spatial condition), and another group completes the RAVLT and SCT (verbal condition).  An a priori power analysis was conducted using G*Power 3.1. The statistical test specified was a z-test for comparing two independent Pearson's r. Assuming a moderate-to-large effect size (Cohen’s q = 0.4), α = 0.05, and power = 0.95, the analysis indicated that a total sample size of N = 166 participants (83 per group) would be sufficient to detect the expected difference in correlations. 

 This estimate was further supported by prior research using similar methodologies. For instance, Costa et al. (2021) validated two immersive VR tasks in a correlational framework with a smaller sample (N = 48), yet reported meaningful associations with traditional spatial measures. Likewise, He et al. (2022) employed a psychometric validation approach in VR using a sample of similar magnitude, also confirming robust convergent validity.  While the power analysis provides an ideal sample size, these prior studies suggest that our methodological approach is feasible and well-grounded in the existing literature, even with smaller samples. We now include this rationale in the manuscript to clarify the reasoning behind our design and effect size assumptions.     

 b) The theoretical motivation for some of the experimental manipulations is unclear. Specifically, it is not clear why the researchers included the experimental manipulation in which half of the participants do NOT complete the spatial test but rather a “non-spatial auditory verbal task to isolate memory from spatial reasoning.” This explanation is inadequate, particularly given the centrality of this manipulation in the experimental design. More information is needed regarding what specifically the alternate task is designed to control for and what construct it is designed to operationalize and measure.   We also acknowledge that the choice of a between-subjects design, rather than a within-subjects one, may warrant further justification. This decision was primarily driven by concerns about carryover effects and interference between tasks. In a within-subjects design, participants would necessarily complete both the spatial and non-spatial tasks, which could lead to contamination across conditions—especially given the possibility that completing one task might influence strategy use, engagement, or performance in the other.    

 Furthermore, since the non-spatial auditory verbal task is intended to serve as a control condition that isolates domain-general memory demands, having participants complete both tasks might undermine the interpretability of the contrast. A between-subjects design avoids these confounds by ensuring that each participant engages with only one cognitive domain, allowing for a cleaner comparison between spatial and non-spatial processing effects.     Finally, the between-subjects design helps maintain ecological validity by mimicking more naturalistic situations in which individuals rely predominantly on either spatial or non-spatial strategies, rather than switching between the two in quick succession.  We considered the possibility of counterbalancing the spatial and non-spatial tasks within a within-subjects design to control for order effects. However, we ultimately decided against this approach for both theoretical and methodological reasons.   

 From a theoretical standpoint, our main goal was to isolate domain-specific contributions to spatial learning by comparing distinct cognitive profiles across individuals. Having participants engage in both spatial and non-spatial tasks—even in counterbalanced order—could introduce unwanted transfer or interference effects, as the cognitive demands of one task might influence performance or strategy use in the other. Counterbalancing controls for average order effects, but it does not eliminate the possibility that completing one task fundamentally alters how the other is approached.    

 Moreover, asking participants to perform both tasks would significantly increase task length and complexity, raising the risk of fatigue, reduced engagement, and cross-task contamination—especially problematic given the immersive and cognitively demanding nature of our virtual reality task. A between-subjects design allowed us to maintain experimental focus and ecological validity without these concerns.    

 In short, while counterbalancing can be useful in many contexts, we felt that in this case, it would not adequately address the core concern: keeping the cognitive domains distinct enough to make meaningful comparisons between spatial and non-spatial contributions.  c) The work does not seem to adequately build on prior work with virtual reality. There are now several publicly available VR-based tests of spatial learning (e.g., Weisberg, S. M., & Newcombe, N. S. (2016)[Refer 1]). Admittedly, many of these tests rely on non-immersive VR, but that alone is not a reason to dismiss them without consideration. What specific weaknesses or limitations of prior research will the proposed test address?   We appreciate the reviewer’s comment and agree that prior work using VR-based tests of spatial learning—such as that by Weisberg & Newcombe (2016)—has made substantial contributions to the field. Our intention was not to dismiss these approaches, but rather to build on them by addressing several of their limitations and extending their applicability.    

 One key limitation of many existing VR-based paradigms, including non-immersive desktop-based environments, is that they often lack the embodied interaction and sensorimotor contingencies present in real-world navigation. These features are increasingly recognized as critical for the formation of robust cognitive maps, particularly in naturalistic spatial learning. Our task leverages immersive VR with free exploration and head-tracking to approximate real-world navigation more closely, which allows us to investigate spatial encoding in ecologically valid conditions while maintaining experimental control.     Furthermore, many existing tasks focus primarily on landmark or route learning, often with limited emphasis on the configurational aspects of spatial memory (i.e., metric relations among multiple locations). Our tool was specifically designed to assess the formation and retrieval of configurational knowledge in a structured yet flexible way, which we believe is a less explored but crucial dimension of spatial cognition.     In addition, prior tasks often do not provide clear separation between spatial reasoning and domain-general memory demands. By including a non-spatial control task, our design aims to disentangle these components, allowing us to better isolate the specific contribution of spatial abilities.     We revised the manuscript to explicitly reference and critically engage with relevant prior work—including Weisberg & Newcombe (2016)—and more clearly articulate the novel contributions and theoretical advancements our task is intended to offer  d) The researchers do not consider possible disadvantages or challenges of the “chair-based perspective.” While the researchers are correct regarding possible advantages, this method may also introduce disadvantages. For example, the vestibular cues associated with moving in a chair may be very different from those involved in walking. Can we assume that the unfamiliar chair-based perspective will provide a valid measure of navigation skill in everyday life experience? Why or why not?   We acknowledge that vestibular and proprioceptive cues involved in real-world navigation—such as those engaged during walking—are not fully replicated in a seated, chair-based setup. However, our approach is grounded in the use of the Spatial Configuration Task (SCT) as a validated and reliable measure of spatial orientation and cognitive map formation. The SCT has been used extensively in prior research to assess spatial abilities without requiring full-body movement, and it is widely accepted in the field as a robust proxy for real-world navigation skills.  Our assumption, therefore, is not that the chair-based setup captures all aspects of real-world navigation, but that it can validly assess key components of spatial cognition—particularly allocentric spatial memory—when used in conjunction with established cognitive measures like the SCT. The correlation between our new task (VSCT) and the SCT serves as a central validation criterion in our study. A meaningful correlation (Cohen’s q ≥ 0.3) with SCT would indicate that the VSCT taps into the same core cognitive abilities, supporting its construct validity despite the more limited sensory input.  Moreover, chair-based navigation is increasingly used in VR-based research on spatial cognition, in part because it allows for standardized procedures across participants, including those with mobility limitations. This makes the paradigm more scalable and inclusive for broader research and potential clinical applications.     That said, we recognize the need for caution when generalizing to real-world navigation. Our task is not intended to fully replicate real-life locomotion, but rather to capture key components of spatial configuration learning under controlled conditions. We will clarify these limitations in the manuscript and suggest future work comparing chair-based and ambulatory VR setups to further assess the generalizability of findings across navigation modalities.  e) Some aspects of the methods are underspecified. Basic information about the test, including the number of trials, is not included. Also, how will the psychometric reliability be assessed? Is there any preliminary data to suggest the test will be reliable? Likewise, the discussion of the expected results seems underspecified. For example, the authors predict positive correlations between some of the measures, but most tests show some positive relationship with each other. Thus, simply showing a positive correlation is not enough to claim that the test has been validated. Therefore, a more specific prediction regarding what level of correlation would be considered evidence of validation is needed.   Thank you for these helpful observations. We agree that some methodological aspects of the task and validation approach were under-specified in the current version of the manuscript, and we appreciate the opportunity to clarify and expand on these elements.  First, we revised the methods section to include more detailed information about the structure of the test, including the number of trials, and trial duration. These details were inadvertently omitted in the current draft and are essential for replicability and for understanding the psychometric potential of the task.     Regarding reliability, we plan to assess internal consistency using split-half and/or Cronbach’s alpha methods for key performance metrics (e.g., accuracy, error scores), depending on distributional characteristics. In addition, we aim to conduct a test–retest reliability study in a subsample of participants to examine stability over time. Although formal reliability estimates are not yet available, preliminary data from pilot testing suggest that performance patterns are consistent across trials and across participants, supporting the potential for good internal reliability.      As for the validation analyses, we agree that simply observing positive correlations with other tasks is insufficient. We will more clearly specify our a priori expectations regarding the magnitude of the correlations that would constitute meaningful evidence of convergent validity. Specifically, we anticipate moderate to strong correlations (e.g., r ≥ .40) with established measures of spatial configuration learning, and weaker correlations with non-spatial control measures. This pattern would support the construct validity of our tool while also demonstrating discriminant validity.  We will revise the manuscript to articulate these expectations explicitly, include benchmarks for interpretation, and outline how we plan to evaluate both convergent and discriminant validity in the context of the broader cognitive framework.  f) One minor issue: Too many abbreviations are used, and they are used too frequently, sometimes within the same sentence. This makes it difficult to follow the text.

# Reviewer report: Reviewer response for version 1

**DOI:** 10.21956/openreseurope.21031.r51668

**Published:** 2025-03-19

**Authors:** Simon Lhuillier

**Affiliations:** 1 Université Gustave Eiffel, Université de Paris, Versailles, France

The aim of this research protocol is to validate a tool for assessing high-level spatial abilities, the principle of which is to improve the ecological validity of a pre-existing 2D tool (Spatial Configuration Task) by implementing an immersive virtual reality headset (Virtual SCT). The usefulness of such a tool seems well justified by the importance of proprioceptive and rotational information involved in the creation of a mental representation of space. However, in its present form, this study has a number of critical flaws which I believe would categorically prevent its publication. However, as this is a pre-protocol study, I consider that substantial changes can still be made to the experimental design and I do not recommend refusing indexed outright, provided that such changes are indeed made. Please find below a summary of the major limitations of the study, followed by minor comments.

**MAJOR COMMENTS** :

- The theoretical justifications for what the task assesses are unclear. What exactly do the authors mean by “cognitive maps” and “spatial representation” ? They introduce a broad definition of spatial abilities but then use the terms “spatial representation” and “cognitive maps” without clearly defining them. This is problematic because multiple theories exist regarding spatial representations, some of which may not be assessable through the task used in the study. Additionally, the authors never define “navigation,” yet it is widely understood to involve goal-directed movement through space (Montello, 2015), which does not apply here : but most models of spatial representation assume a reliance on neural mechanisms (as described in the first author’s thesis) that are unlikely to be involved in the VSCT—such as place cells and grid cells, which contribute to egocentric spatial binding in navigation. Beyond these two broad notions, the study provides no further details on the specific spatial abilities relevant to the VSCT—such as mental rotation or perspective-taking. Furthermore, the discussion omits key concepts, such as whether participants rely on an egocentric or allocentric frame of reference to solve the task, which seems crucial to consider.

 

- The research design does not seem well-suited to address the study’s objective. The authors claim that VR “offers several advantages over traditional 2D and 3D desktop assessments of spatial abilities” (including over the SCT), yet they do not plan to directly compare SCT and VSCT. Instead, they use the VSCT as a training method for an SCT retest. While this approach does not allow for validating the VSCT as a spatial ability test, the choice of a comparison condition—a non-spatial task (RAVLT)—also limits the ability to assess the effectiveness of VSCT training. A proper evaluation would require a comparison not only with a non-spatial control condition but also with a non-VR spatial training condition. Moreover, the authors do not justify why they expect performing a VR task to facilitate the SCT retest. Without a clear rationale, one might assume that the VSCT is simply another iteration of the SCT, serving as direct practice for the SCT task rather than training the spatial abilities it is supposed to measure. The introduction to the discussion further highlights this conceptual confusion: the authors state that their goal is to validate the VSCT for assessing cognitive map formation, yet the VSCT is never actually used to evaluate this ability in the study. Additionally, alternative tests that could serve as comparisons (e.g., Gehrke et al., 2018; Hartman et al., 2006) are mentioned but not directly compared to the VSCT.

 

- The analysis plan is not appropriate. First, age and sex are treated as independent variables, yet they are never discussed in the introduction. All independent variables should be thoroughly justified based on existing literature in the introduction and commented in the discussion. Additionally, given the study design, the independent variables must include the between-subject experimental condition (VR vs. AV) and account for within-subject changes in SCT scores before and after training. A proper repeated-measures analysis should be used to analyse these effects.

**MINOR COMMENTS** :

Introduction

- Page 3 : Extra parenthesis after (Bartlett & Camba, 2022).
- Page 3 : Authors argue that a VR implementation of the SCT is needed because physically rotating to visually explore the environment should reduce motion sickness. This claim is highly disputable regarding scientific literature, as articles and reviews on the matter suggest that HMD are in fact producing more motion sickness symptoms than other displays (see Rebenitsch & Owen, 2016).

Method

- Page 4 : More details are needed regarding the sample size calculation. The specific analysis for which the calculation was performed must be explicitly stated (i.e., the "type of power analysis" used in G*Power). As it stands, the reported effect size (f) suggests that an incorrect type of power analysis was used, given that f is used for ANOVA, while no ANOVA is planned in the study. Additionally, a sample size of n=54 for two independent groups appears rather low, justifying this by citing a single other study with a similar sample size is insufficient—especially since He et al. (2022) used a fully within-subject design.
- Page 4 : The “screening phase” is very confusing. It is mentioned, but no inclusion/exclusion criteria are reported. Moreover, it is said after that the participants will be assessed online via Psytoolkit : is it still screening phase ? Why are both Psytoolkit and Qualtrics used ? Why are there “psychometric tests” during screening and which one ?
- Page 4 : I don’t understand why this separate sentence “the spatial performances […] following tasks” is isolated before describing a non-spatial task (RAVLT) a paragraph later.
- Page 4 : The SCT is a task administered during the study, so it must be described in detail (regardless of whether it has been published previously). In addition, all differences between SCT and VSCT should be explicitly listed so that readers can directly understand what differs between SCT and VSCT.
- Page 4 : How the SSQ is used is not clear, as it is likely not used to compare motion sickness in the SCT vs VCST (which would yet be useful here to check for increased risk of motion sickness with VR). It is not referred to in the analysis plan section.
- Page 4 : An eye tracking system is mentioned here, but no ocular data is described in the analysis plan.
- Page 5 : The justification for changing geometrical shapes for objects in VSCT seems shallow, as the resulting scenario cannot be considered as “immersive” and “ecologically valid” because of the settings (empty space with random objects on the ground, and not a “room” as stated).
- Page 5 : It is unclear if the objects are the same in the 10 different scenes, and what exactly is different between all of them ?
- Page 5 : Why are there “missing objects” during the training phase ?
- Page 5 : Training phase section is misleading. Why would participants return to the main menu, and switch scenes by themselves, why choosing 20 seconds maximum time, and why 20 scenes while authors said that only 10 scenes were created ? Do participants answer spatial task (like in Figure 1a) during this training phase ?
- Page 5 : The title “Environment navigation/locomotion” is misleading. Is locomotion involved or not ? Authors specifically said before that participants only rotated on a chair.
- Page 5 and 6 : Figures should be higher resolution
- Page 6 : the procedure suggests that the training phase is in fact a learning phase, followed by a test phase. Needs clarification.
- Page 6 : Writing style should be inclusive (“(two in front of them, and one behind them”)).
- Page 6 : Why only three consecutive trials ? Isn’t it very short and easy ? If not please provide more information.
- Page 6 : about the RAVLT, “it is crucial to present the words in the same order and with the same pronunciation for each participants”. Why not using audio recording or a video for this ?
- Page 6 : The scrambled image test is never described
- Page 6 : “ This will help us understand the influence of global intelligence and working memory capacity”. No general intelligence test is mentioned elsewhere in the study

Discussion

- Page 7 : Potential applications of the VSCT for populations with impaired movement. You need to specify which specific populations you are referring to.
- Page 7 : “The task’s reliance on proprioceptive feedback […] enhances its effectiveness in spatial learning and navigation”. You don’t and will not know this, since no direct comparison is made with SCT
- The global tone must be toned down, no data are collected and such conclusions would not be permitted with the chosen design : “the validation of the VSCT represents a significant advancement in spatial cognition research”

Is the study design appropriate for the research question?

No

Is the rationale for, and objectives of, the study clearly described?

Partly

Are sufficient details of the methods provided to allow replication by others?

No

Are the datasets clearly presented in a useable and accessible format?

Not applicable

Reviewer Expertise:

Spatial cognition, spatial representations, virtual reality, spatial and temporal cognitive interactions, embodied and situated cognition

I confirm that I have read this submission and believe that I have an appropriate level of expertise to state that I do not consider it to be of an acceptable scientific standard, for reasons outlined above.

## References

[1] : Spatial Cognition.2015; 10.1016/B978-0-08-097086-8.72057-5 111-115 10.1016/B978-0-08-097086-8.72057-5

[2] : Review on cybersickness in applications and visual displays. *Virtual Reality* .2016;20(2) : 10.1007/s10055-016-0285-9 101-125 10.1007/s10055-016-0285-9

# Response

**Published:** 2025-04-26

**Authors:** Giorgio Li Pira

**Affiliations:** Psicologia, Universita degli Studi di Bologna Dipartimento di Psicologia Renzo Canestrari, Bologna, Emilia-Romagna, Italy

**MAJOR COMMENTS : ** 

                     The theoretical justifications for what the task assesses are unclear. What exactly do the authors mean by “cognitive maps” and “spatial representation”? They introduce a broad definition of spatial abilities but then use the terms “spatial representation” and “cognitive maps” without clearly defining them. This is problematic because multiple theories exist regarding spatial representations, some of which may not be assessable through the task used in the study. Additionally, the authors never define “navigation,” yet it is widely understood to involve goal-directed movement through space (Montello, 2015), which does not apply here: but most models of spatial representation assume a reliance on neural mechanisms (as described in the first author’s thesis) that are unlikely to be involved in the VSCT—such as place cells and grid cells, which contribute to egocentric spatial binding in navigation. Beyond these two broad notions, the study provides no further details on the specific spatial abilities relevant to the VSCT—such as mental rotation or perspective-taking. Furthermore, the discussion omits key concepts, such as whether participants rely on an egocentric or allocentric frame of reference to solve the task, which seems crucial to consider.    We agree that terms like cognitive map, spatial representation, and navigation can carry multiple theoretical meanings, and it is essential to define how we are using them within the context of this study. In response to your comment, we have now explicitly defined a cognitive map as a mental representation of the spatial layout of an environment, allowing an individual to acquire, code, store, recall, and decode information about the relative locations of places, landmarks, and routes [(Tolman, 1984)].  In addition, we have added a paragraph in the introduction, elaborating on the theoretical background surrounding allocentric and egocentric spatial strategies, both of which are central to the concept of cognitive mapping. Specifically, we acknowledge that allocentric strategies involve representing spatial relationships between landmarks independently of the observer’s position (i.e., map-like), whereas egocentric strategies encode spatial information relative to the observer’s own viewpoint.  While the VSCT does not involve full bodily movement or large-scale wayfinding, it is designed to assess an individual’s ability to mentally represent and recall the spatial relationships among landmarks within a 3D virtual environment. This setup supports allocentric encoding by allowing participants to build a representation of the layout across different viewpoints and potentially engages egocentric processing during the encoding phase. Thus, although the task may not elicit neural activity from systems such as grids or place cells in the same way real-world navigation does, it targets higher-order spatial abilities that are widely accepted as central components of spatial cognition and cognitive map formation.    The research design does not seem well-suited to address the study’s objective. The authors claim that VR “offers several advantages over traditional 2D and 3D desktop assessments of spatial abilities” (including over the SCT), yet they do not plan to directly compare SCT and VSCT. Instead, they use the VSCT as a training method for an SCT retest. While this approach does not allow for validating the VSCT as a spatial ability test, the choice of a comparison condition—a non-spatial task (RAVLT)—also limits the ability to assess the effectiveness of VSCT training. A proper evaluation would require a comparison not only with a non-spatial control condition but also with a non-VR spatial training condition. Moreover, the authors do not justify why they expect performing a VR task to facilitate the SCT retest. Without a clear rationale, one might assume that the VSCT is simply another iteration of the SCT, serving as direct practice for the SCT task rather than training the spatial abilities it is supposed to measure. The introduction to the discussion further highlights this conceptual confusion: the authors state that their goal is to validate the VSCT for assessing cognitive map formation, yet the VSCT is never actually used to evaluate this ability in the study. Additionally, alternative tests that could serve as comparisons (e.g., Gehrke et al., 2018; Hartman et al., 2006) are mentioned but not directly compared to the VSCT.      We appreciate the opportunity to clarify our research design and the rationale behind it.  Our study attempts to directly compare the newly developed VSCT and the SCT at Time 1 (T1). Specifically, we run the comparison between VSCT performance and SCT scores to assess convergent validity and to fulfil our primary objective, i.e., to determine whether the VSCT reliably measures cognitive map formation abilities, as operationalized through an established spatial task.  On the contrary, we acknowledge that the SCT retest was not designed to serve as a formal validation criterion. Rather, we have positioned the SCT retest as an exploratory component—intended to evaluate whether performing the VSCT might influence spatial recall when the SCT is administered again. This secondary analysis aims to explore potential transfer effects, which are ancillary to our primary validity measures.  As for the use of a non-spatial task (RAVLT) in the comparison group, our intention is not to measure training effectiveness, but to test discriminant validity. By comparing the correlation between SCT and VSCT (spatial-spatial pairing) versus SCT and RAVLT (spatial-verbal pairing), we can determine whether VSCT is specifically related to spatial abilities rather than general memory performance.  We agree that additional comparison conditions—such as non-VR spatial tasks or direct comparisons to alternative paradigms like those proposed by Gehrke et al. (2018) or Hartman et al. (2006)—would provide further insight. However, this study focuses on the initial validation of the VSCT as a spatial cognition tool and lays the groundwork for future work that will include those broader comparisons.  We will clarify these points in the revised manuscript to make our rationale and design choices more transparent.     The analysis plan is not appropriate. First, age and sex are treated as independent variables, yet they are never discussed in the introduction. All independent variables should be thoroughly justified based on existing literature in the introduction and commented in the discussion. Additionally, given the study design, the independent variables must include the between-subject experimental condition (VR vs. AV) and account for within-subject changes in SCT scores before and after training. A proper repeated-measures analysis should be used to analyse these effects.  Age and sex are not treated as independent variables in our primary analyses. They were included for the mere purpose of ensuring that the two experimental groups (VR vs. verbal task) are demographically balanced, not to examine their effects on task performance. As such, we did not discuss them in the introduction or interpret them in the discussion, as they are not central to our research questions.  Regarding the repeated-measures design, we appreciate your suggestion. However, our primary objective is not to assess the training effectiveness of the VSCT but rather to validate it as a tool for measuring cognitive map formation. The SCT retest is included as an exploratory follow-up, not as a main outcome, and therefore was not incorporated into our preregistered statistical plan. Since the validation is based on cross-sectional correlations (e.g., VSCT ~ SCT at T1) and group comparisons in convergent vs. discriminant validity, a repeated-measures analysis of SCT scores was not considered essential to address the core aims of the study.  We acknowledge the potential value of further exploring training effects in future work and will clarify this distinction in the manuscript to avoid confusion about the study’s primary focus. Thank you again for helping us improve the clarity and rigor of our design and reporting.    MINOR COMMENTS :     Introduction    

- Page 3 : Extra parenthesis after (Bartlett & Camba, 2022). 

Typo corrected  Page 3 : Authors argue that a VR implementation of the SCT is needed because physically rotating to visually explore the environment should reduce motion sickness. This claim is highly disputable regarding scientific literature, as articles and reviews on the matter suggest that HMD are in fact producing more motion sickness symptoms than other displays (see Rebenitsch & Owen, 2016).    We acknowledge that head-mounted displays (HMDs) can, in some contexts, be associated with motion sickness, we respectfully disagree with the interpretation that this undermines the rationale for our design. Specifically, our claim is not that HMDs universally reduce motion sickness compared to all display types, but that using real physical movement (i.e., body rotation) within an HMD setup can significantly reduce motion sickness when compared to artificial locomotion methods such as joystick or controller-based movement.  This distinction is critical. Numerous studies have demonstrated that sensorimotor congruence—where physical movements of the body align with the visual flow in the VR environment—reduces cybersickness symptoms. Passive or controller-based movement often introduces sensory conflict, which is a well-established contributor to motion sickness in VR. By enabling users to physically rotate to explore the virtual environment (as in our task), we aim to minimize sensory mismatch and thus mitigate motion sickness.  Furthermore, the objective of our task is to validate spatial cognition within immersive VR environments, not to compare HMDs to non-immersive or 2D display formats. Our interest is specifically in how to optimize immersion within HMD-based tasks while minimizing discomfort—not in comparing display technologies, which falls outside the scope of this study.    Method   Page 4 : More details are needed regarding the sample size calculation. The specific analysis for which the calculation was performed must be explicitly stated (i.e., the "type of power analysis" used in G*Power). As it stands, the reported effect size (f) suggests that an incorrect type of power analysis was used, given that f is used for ANOVA, while no ANOVA is planned in the study. Additionally, a sample size of n=54 for two independent groups appears rather low, justifying this by citing a single other study with a similar sample size is insufficient—especially since He et al. (2022) used a fully within-subject design.  Thank you for your careful reading and constructive comment. We fully agree that the research question and the focus of the power analysis must be clearly and explicitly stated. We have revised the manuscript accordingly to clarify both the analytical approach and the rationale for the sample size.  To clarify: the main objective of the study is to test the construct validity of the newly developed Virtual Spatial Configuration Task (VSCT), by assessing:  Convergent validity through correlations between VSCT and two established spatial cognition measures (Spatial Configuration Task [SCT] and Corsi Block-Tapping Task), and  Discriminant validity by comparing the correlation between SCT and VSCT with that between SCT and a verbal memory task (Rey Auditory Verbal Learning Test [RAVLT]).  To test discriminant validity directly, we employ a between-subjects design in which:  One group completes the VSCT and SCT (spatial condition),  Another group completes the RAVLT and SCT (verbal condition).  We conducted an a priori power analysis using G*Power 3.1, specifying the statistical test as a z-test comparing two independent Pearson’s r values (i.e., correlation coefficients). The analysis assumed a moderate-to-large effect size (Cohen’s q = 0.4), α = .05, and power (1 – β) = 0.95. The required sample size for this test was N = 166 (i.e., 83 per group). This calculation has now been explicitly described in the Methods section of the revised manuscript.  The choice of effect size was informed by Cohen’s (1988) conventions, but also weighted by empirical precedent from previous studies. For instance, Costa et al. (2021) used a similar immersive VR setting and demonstrated significant convergent correlations using a comparable sample. Furthermore, He et al. (2022) validated a spatial perspective-taking task using a within-subjects design and a similar sample size. However, we acknowledge—as the reviewer rightly points out—that within-subjects designs generally offer higher statistical power than between-subjects designs. We now explicitly note this difference in the revised manuscript and justify our more conservative effect size assumption by accounting for the increased variability introduced by immersive VR environments, which typically require additional training and participant adaptation.       

- Page 4 : The “screening phase” is very confusing. It is mentioned, but no inclusion/exclusion criteria are reported. Moreover, it is said after that the participants will be assessed online via Psytoolkit : is it still screening phase ? Why are both Psytoolkit and Qualtrics used ? Why are there “psychometric tests” during screening and which one ? 

Thank you for pointing out the confusion regarding the terminology and procedures described on Page 4.  To clarify, we have updated the term “screening phase” to “first evaluation phase” to better reflect the nature of this initial step. This change was made to avoid the implication that formal inclusion or exclusion criteria were being applied.  In this first evaluation phase, we do not apply any exclusion criteria. The purpose of this phase is simply to collect demographic information and participants’ performance on the Corsi Block-Tapping Task, which is used later for correlational analyses in the validation process.  Regarding the use of PsyToolkit and Qualtrics: 

- PsyToolkit was used to administer the Corsi task, as it provides a validated and widely used online implementation. 

- Qualtrics was used to collect demographic data (e.g., age, sex). 

There are no additional psychometric tests administered in this phase. We will revise the manuscript accordingly to clearly describe this procedure and prevent any further confusion. Thank you again for your helpful feedback.   

- Page 4 : I don’t understand why this separate sentence “the spatial performances […] following tasks” is isolated before describing a non-spatial task (RAVLT) a paragraph later. 

We specified that we used RAVLT not to test spatial performances but as discriminat measure of verbal vs spatial memory   We agree that the sentence placement may have caused some confusion. To clarify, the sentence referring to “spatial performances” was intended to introduce the tasks specifically used to assess spatial abilities, such as the VSCT, SCT, and Corsi.  We have now clarified in the manuscript that the RAVLT was not included to assess spatial performance, but rather used as a discriminant measure—to differentiate verbal memory from spatial memory processes. This distinction supports the assessment of discriminant validity, by allowing us to test whether the newly developed spatial task (VSCT) is more strongly associated with spatial rather than verbal cognitive functions.  We have revised the paragraph to improve the logical flow and ensure the role of each task is clearly described.    Page 4 : The SCT is a task administered during the study, so it must be described in detail (regardless of whether it has been published previously). In addition, all differences between SCT and VSCT should be explicitly listed so that readers can directly understand what differs between SCT and VSCT.  While we acknowledge that the description of the Spatial Configuration Task (SCT) is brief, our primary focus in this manuscript is the development and validation of the Virtual Spatial Configuration Task (VSCT). The SCT has been thoroughly described and validated in the referenced publication (Burles et al., 2017), which we cite for readers seeking detailed methodological information. Given the scope of this study, we chose not to include redundant details and instead directed our attention to the novel aspects of the VSCT.    Page 4 : How the SSQ is used is not clear, as it is likely not used to compare motion sickness in the SCT vs VCST (which would yet be useful here to check for increased risk of motion sickness with VR). It is not referred to in the analysis plan section.  We thank the reviewer for this observation and would like to clarify the role of the Simulator Sickness Questionnaire (SSQ) in our study. While the Spatial Configuration Test (SCT) is indeed computer-based, it is not immersive and does not involve simulated self-motion, 3D depth cues, or head tracking. As such, it does not typically elicit motion sickness symptoms, and we do not expect it to trigger the kind of sensory mismatch effects associated with immersive VR. For this reason, the SSQ was administered only in the context of the Virtual Spatial Configuration Task (VSCT), which is immersive and includes active visual exploration through head movements. Our intention was not to use the SSQ for cross-condition comparisons, but rather as a monitoring tool to ensure participant comfort and to identify any individuals who experienced significant motion sickness in the VR condition.  Page 4 : An eye tracking system is mentioned here, but no ocular data is described in the analysis plan.  You are correct that the mention of the eye-tracking system was potentially misleading. It was originally included simply to provide a more complete description of the hardware setup used during the task administration, but we confirm that no ocular data were recorded or analyzed as part of this study.  To avoid confusion, we have now removed this detail from the manuscript, since it is not relevant to the research questions or analysis plan.     Page 5 : The justification for changing geometrical shapes for objects in VSCT seems shallow, as the resulting scenario cannot be considered as “immersive” and “ecologically valid” because of the settings (empty space with random objects on the ground, and not a “room” as stated).  Thank you for this helpful suggestion. We agree with the reviewer that, given the minimalistic and artificial nature of the virtual environment, referring to the use of household objects as enhancing "ecological relevance" may have been misleading. Instead, we have revised the text to emphasize that these objects were chosen for their high familiarity and recognizability, which helps reduce variability in object encoding and supports more consistent performance across participants. This change has been reflected in the revised manuscript accordingly. 

   Page 5 : It is unclear if the objects are the same in the 10 different scenes, and what exactly is different between all of them?    Page 5 : Why are there “missing objects” during the training phase ?  We have now eliminated the training phase from the protocol and updated the task section in accordance  Page 5 : Training phase section is misleading. Why would participants return to the main menu, and switch scenes by themselves, why choosing 20 seconds maximum time, and why 20 scenes while authors said that only 10 scenes were created ? Do participants answer spatial task (like in Figure 1a) during this training phase ?  See comment above    Page 5 : The title “Environment navigation/locomotion” is misleading. Is locomotion involved or not? Authors specifically said before that participants only rotated on a chair.  We thank the reviewer for pointing this out. We agree that the term “locomotion” was misleading, as participants did not physically navigate through space but rather explored the virtual environment by rotating on a chair. To better reflect the actual procedure, we have replaced “locomotion” with “exploration” throughout the manuscript, including in the section title. This change ensures terminological accuracy and avoids confusion regarding the nature of participants’ movements during the task.  Page 5 and 6 : Figures should be higher resolution  We will upload better resolution images  Page 6 : the procedure suggests that the training phase is in fact a learning phase, followed by a test phase. Needs clarification.  We have now eliminated the training phase from the protocol and updated the task section in accordance    Page 6 : Writing style should be inclusive (“(two in front of them, and one behind them”)).  We appreciate the reviewer’s suggestion regarding inclusive writing. We have revised the relevant sentence—and others where needed—to ensure a more inclusive and neutral phrasing (e.g., replacing “in front of them”/“behind them” with spatially clear, participant-centered language). We thank the reviewer for drawing our attention to this.  We appreciate the reviewer’s suggestion regarding inclusive writing. We have revised the relevant sentence—and others where needed—to ensure a more inclusive and neutral phrasing (e.g., replacing “in front of them”/“behind them” with spatially clear, participant-centered language). We thank the reviewer for drawing our attention to this.     We appreciate the reviewer’s suggestion regarding inclusive writing. We have revised the relevant sentence—and others where needed—to ensure a more inclusive and neutral phrasing (e.g., replacing “in front of them”/“behind them” with spatially clear, participant-centered language). We thank the reviewer for drawing our attention to this.  Page 6 : Why only three consecutive trials ? Isn’t it very short and easy ? If not please provide more information.  The task has since been modified: there is no longer a training phase. Participants are now required to complete 40 trials in the same manner as the SCT.  Page 6 : about the RAVLT, “it is crucial to present the words in the same order and with the same pronunciation for each participants”. Why not using audio recording or a video for this?  We thank the reviewer for this suggestion. While we agree that audio or video recordings can enhance standardization, in this case we chose to follow the official administration guidelines for the RAVLT, which specify live verbal presentation by an examiner. To ensure consistency, the same trained experimenter administered the word lists to all participants, strictly following the standardized timing and pronunciation provided in the RAVLT manual. This approach preserves both the test’s ecological and methodological validity, as it aligns with how the RAVLT is commonly used and interpreted in clinical and research settings.   Page 6 : The scrambled image test is never described  You're absolutely right—the scrambled image test was not designed as a cognitive measure and has no analytical role in our study. Its sole purpose was to serve as a neutral filler task to occupy participants during the delay interval between the encoding and recall phases of the VSCT.  To avoid confusion, we have now clarified this point in the manuscript and explicitly stated that the scrambled image task is included only to control for retention interval duration, with no data recorded or analyzed from this task.    Page 6 : “ This will help us understand the influence of global intelligence and working memory capacity”. No general intelligence test is mentioned elsewhere in the study    You are correct — no general intelligence measure is included in the study. That sentence originally reflected an earlier version of the design.  We have since removed the reference to “global intelligence” and now refer only to working memory capacity, as assessed by the Corsi Block-Tapping Task. The sentence has been revised accordingly to accurately reflect the current design and avoid any confusion about the variables being examined.  Discussion     Page 7 : Potential applications of the VSCT for populations with impaired movement. You need to specify which specific populations you are referring to.  We appreciate the reviewer’s request for clarification. Our reference to populations with impaired movement was meant to suggest the potential applicability of the VSCT in contexts where full-body locomotion is not feasible. We will revise the manuscript to include illustrative examples—such as individuals with spinal cord injury, neurodegenerative diseases (e.g., multiple sclerosis), or age-related mobility impairments—while avoiding an exhaustive list. The core idea is that the chair-based design of the VSCT allows for the assessment of spatial abilities in individuals who cannot participate in walking-based or ambulatory VR tasks.    Page 7 : “The task’s reliance on proprioceptive feedback […] enhances its effectiveness in spatial learning and navigation”. You don’t and will not know this, since no direct comparison is made with SCT  To clarify, our intention was to highlight that, unlike the SCT, the VSCT allows for physical rotational movement, which—according to existing literature—can engage vestibular and proprioceptive systems, both of which are known to play a role in spatial orientation and learning. While we do not directly measure the contribution of these sensory inputs in this study, we base this aspect of our design on prior evidence suggesting that physical movement, particularly head and body rotation, can enhance spatial processing by reducing sensory mismatch and engaging real-world navigation systems.  The global tone must be toned down, no data are collected and such conclusions would not be permitted with the chosen design : “the validation of the VSCT represents a significant advancement in spatial cognition research”  We fully agree with your point—since no empirical data have yet been collected, strong conclusive statements are premature and not appropriate at this stage.  We have revised the statement to reflect a more cautious and accurate tone. Specifically, we have changed:  “the validation of the VSCT represents a significant advancement in spatial cognition research”  to:  “the validation of the VSCT could represent a meaningful advancement in spatial cognition research”  This adjustment ensures that our claims remain appropriately tentative and aligned with the current study design and preregistration status. We appreciate your guidance in helping us maintain scientific rigor.

# Reviewer report: Reviewer response for version 1

**DOI:** 10.21956/openreseurope.21031.r51674

**Published:** 2025-03-18

**Authors:** Laura Miola

**Affiliations:** 1 University of Padova, Padua, Italy

I reviewed the *Pre-Protocol of the Virtual Spatial Configuration Task (VSCT): A Novel Virtual Reality-Based Tool for Assessing Cognitive Map Formation Abilities*. The study presents the validation of a 3D spatial task designed to evaluate the ability to construct a mental representation of the environment. Overall, the protocol is well-structured, the rationale is clearly articulated, emphasizing the limitations of traditional 2D spatial tasks and the potential benefits of an immersive 3D VR environment. Here are some points that could improve the clarity of the study:

- Since this is a validation study, the authors should explicitly specify how they plan to assess the validity and reliability of the task. While they mention conducting certain analyses, it is unclear which ones are specifically intended to evaluate aspects such as convergent and discriminant validity or reliability. Providing more detail on the methodological approach for these assessments would strengthen the study. Some useful suggestions can be found in the paper: Brysbaert, M. (2024). Designing and evaluating tasks to measure individual differences in experimental psychology: A tutorial. Cognitive Research: Principles and Implications, 9(1), 11.[Refer 1].
- The reasoning behind dividing participants into two groups could be explained more clearly. It would be helpful to elaborate further on why this specific comparison was chosen and how it contributes to the validation process. Additionally, specifying whether the study follows a between-subjects or within-subjects design would help improve clarity.
- I suggest providing a more detailed description of the power analysis, including the statistical analysis used to calculate it. The authors may prefer using Cohen's d or providing a justification for the effect size.
- Although the task closely resembles the Spatial Configuration Task, the description of the training phase is not entirely clear, primarily due to the poor visibility of the images 1c and 1d. Additionally, it is unclear which movements participants were allowed to perform and at what stage.
- In the introduction, I suggest citing theoretical models to define spatial abilities rather than referencing a master's thesis.
- In the hypothesis section, the authors mention the RAVLT task using its acronym without having introduced it earlier. Conversely, the SSQ is fully cited in the introduction, but only its acronym is mentioned in the Methods and Materials section.
- In the linear model analysis, are the authors referring to VSCT or SCT?
- There are some typographical errors related to parentheses in the introduction.

Is the study design appropriate for the research question?

Partly

Is the rationale for, and objectives of, the study clearly described?

Yes

Are sufficient details of the methods provided to allow replication by others?

Partly

Are the datasets clearly presented in a useable and accessible format?

Not applicable

Reviewer Expertise:

Spatial cognition, individual differences, environmental psychology

I confirm that I have read this submission and believe that I have an appropriate level of expertise to confirm that it is of an acceptable scientific standard, however I have significant reservations, as outlined above.

## References

[1] : Designing and evaluating tasks to measure individual differences in experimental psychology: a tutorial. *Cogn Res Princ Implic* .2024;9(1) : 10.1186/s41235-024-00540-2 11 10.1186/s41235-024-00540-2 38411837 PMC10899130

# Response

**Published:** 2025-04-26

**Authors:** Giorgio Li Pira

**Affiliations:** Psicologia, Universita degli Studi di Bologna Dipartimento di Psicologia Renzo Canestrari, Bologna, Emilia-Romagna, Italy

I reviewed the Pre-Protocol of the Virtual Spatial Configuration Task (VSCT): A Novel Virtual Reality-Based Tool for Assessing Cognitive Map Formation Abilities. The study presents the validation of a 3D spatial task designed to evaluate the ability to construct a mental representation of the environment. Overall, the protocol is well-structured, the rationale is clearly articulated, emphasizing the limitations of traditional 2D spatial tasks and the potential benefits of an immersive 3D VR environment. Here are some points that could improve the clarity of the study:     Since this is a validation study, the authors should explicitly specify how they plan to assess the validity and reliability of the task. While they mention conducting certain analyses, it is unclear which ones are specifically intended to evaluate aspects such as convergent and discriminant validity or reliability. Providing more detail on the methodological approach for these assessments would strengthen the study. Some useful suggestions can be found in the paper: Brysbaert, M. (2024). Designing and evaluating tasks to measure individual differences in experimental psychology: A tutorial. Cognitive Research: Principles and Implications, 9(1), 11.[Refer 1].  We appreciate the reviewer’s comment and have revised the manuscript to clarify our approach to evaluating both the validity and reliability of the Visual Spatial Configuration Task (VSCT). Specifically, we now explicitly distinguish between the methods used to assess convergent and discriminant validity, as well as reliability.  To assess discriminant validity, we included the Rey Auditory Verbal Learning Test (RAVLT), not because we anticipated a correlation, but precisely because we expected no significant relationship. The RAVLT measures verbal episodic memory, while the VSCT targets visuospatial configuration memory—two theoretically and empirically distinct constructs. The absence of a significant correlation between these tasks supports the discriminant validity of the VSCT by demonstrating that it does not simply reflect general memory ability.  In addition, we have now outlined our plans for assessing convergent validity, which involve correlating performance on the VSCT with other tasks that tap into similar visuospatial memory constructs (details included in the updated Methods section, P3).  Regarding reliability, we are preparing a second study since the first one involved only a one-time measurement of VSCT. In the next study, we will expose participants to VSCT one week apart to assess test-retest reliability.  These clarifications have been incorporated into the revised manuscript (see P3), strengthening the methodological transparency of the study.  The reasoning behind dividing participants into two groups could be explained more clearly. It would be helpful to elaborate further on why this specific comparison was chosen and how it contributes to the validation process. Additionally, specifying whether the study follows a between-subjects or within-subjects design would help improve clarity.  Thank you for your helpful comment. In response, we have clarified the reasoning behind dividing participants into two groups and specified the study design. The revised text now reads:  “This study aims to validate the newly developed VSCT by evaluating participants' performance on the original SCT both before and after VR exposure. We will use a between-subjects design, sorting participants into two groups to clarify the specific effects of spatial versus non-spatial cognitive demands. One group will perform the task in virtual reality, while the other will engage in a non-spatial auditory verbal task, adapted from Bacon et al. (2008), to isolate memory processes from spatial reasoning. This comparison allows us to determine whether improvements or changes in SCT performance are specifically related to spatial cognitive training, rather than general cognitive engagement. Both groups will also complete standardized cognitive tests targeting functions like working memory, which are involved in constructing cognitive maps, to further assess the VSCT's validity as a spatial cognition tool.”  We hope this revision addresses your concerns and improves the clarity and rationale of our study design.    I suggest providing a more detailed description of the power analysis, including the statistical analysis used to calculate it. The authors may prefer using Cohen's d or providing a justification for the effect size.  Thank you for your suggestion.     We have revised the manuscript to include a more detailed description of the power analysis, including the statistical model used and the justification for the chosen effect size.    Although the task closely resembles the Spatial Configuration Task, the description of the training phase is not entirely clear, primarily due to the poor visibility of images 1c and 1d. Additionally, it is unclear which movements participants were allowed to perform and at what stage.  Thank you for your observation. We have revised the text to clarify the type of movement allowed during the task. Specifically, we now state: 

 “In our study, translational movement is not performed by participants. Instead, participants are seated in a swivel chair that allows them to actively rotate their torso left and right, enabling 360-degree exploration of the environment.”  Regarding the figure, we acknowledge that the resolution of images 1c and 1d in the uploaded protocol is suboptimal. We believe this issue may be due to image degradation during the online conversion process. To address this, we will upload a higher-resolution version of the figure to ensure better visibility and clarity.  In the introduction, I suggest citing theoretical models to define spatial abilities rather than referencing a master's thesis.  Thank you for your suggestion. We understand the importance of grounding definitions in well-established theoretical models. However, because our work builds directly on the Spatial Configuration Task (SCT), we felt it was meaningful to reference the master’s thesis that contributed to the initial validation of the SCT. This citation acknowledges the foundational work related to the task’s development and helps contextualize our adaptation. That said, we appreciate your point and included additional references to broader theoretical models of spatial abilities to complement this citation.  In the hypothesis section, the authors mention the RAVLT task using its acronym without having introduced it earlier. Conversely, the SSQ is fully cited in the introduction, but only its acronym is mentioned in the Methods and Materials section.  Thank you for pointing this out. We have corrected the oversight by introducing the full name of the RAVLT (Rey Auditory Verbal Learning Test) before using its acronym. Regarding the SSQ (Simulator Sickness Questionnaire), we believe it is sufficient to use the acronym after it has been fully cited in the introduction, in line with standard academic conventions.  In the linear model analysis, are the authors referring to VSCT or SCT?  We have slightly modified the analysis plan to better clarify the purpose of the measures described. Specifically, we now state that the SCT is the outcome variable used in the t-test and linear regression analyses. These analyses aim to assess potential differences between the control and experimental groups and to verify whether age and sex—which were collected as sociodemographic variables known to influence spatial navigation—could act as confounders. Additionally, we now specify that independent samples t-tests will also be conducted to compare CORSI and SBSOD scores between groups, in order to further confirm group equivalence on relevant cognitive measures. These revisions have been made to enhance clarity and align the text with the objectives of the study.  There are some typographical errors related to parentheses in the introduction.  Mistake corrected.

# Reviewer report: Reviewer response for version 1

**DOI:** 10.21956/openreseurope.21031.r51672

**Published:** 2025-03-06

**Authors:** Julian Keil

**Affiliations:** 1 Ruhr University Bochum, Bochum, Germany

The study protocol of Alberto Massimiliano Umiltà and colleagues proposes an approach to validate a task aimed to assess spatial memory and performance. The authors describe the disadvantages of assessing spatial abilities using commonly applied 2D tasks very well. They provide an interesting alternative 3D task created in Unity and using an VR HMD. The selection of sample size and the sampling process are well described. However, important details on the experiment design, hypotheses and the experiment procedure are still missing. These details are required to fully evaluate the appropriateness of the suggested spatial task for its intended purpose.

**General comments:**

 The cited Burles F and Burles CF are probably the same person (Ford Burles)? Please check your references and citations again for potential discrepancies.

 I recommend that you look into the work of Stangl and colleagues (https://doi.org/10.1016/j.cub.2018.02.038). They used an approach that has many similarities to the approach suggested in your manuscript.

 The HTC Vive Pro Eye is a good HMD in terms of spatial tracking, display resolution and eye tracking. However, you should consider also using the HTC Vive Wireless Adapter. It allows users to rotate without paying attention to the cable, which can affect immersion and task performance. The wireless adapter can be used without any changes to the experiment design and (in my experience) does not add any noticeable latency.

 Some details of the experiment are not sufficiently described: What is the task in the training phase and how does the training phase end? Are the locations of the (visible and invisible) objects always the same? Do the participants receive feedback concerning their response? If not, how do they learn the spatial configuration if only two objects are visible simultaneously? How are performance scores on the VSCT calculated?

 It remains unclear what the added benefit of the VSCT compared to the SCT is. If you expect the results on both tests to correlate, why would you choose the more resource intensive and VR-sickness-prone VSCT? Maybe, you could extend you proposed statistical analysis to investigate potential advantages of the VSCT over the SCT.

 The described approach of data and code publication is appropriate to ensure accessibility and reproducibility.

**Detailed comments:**

 P3: “Furthermore, 2D tests do not allow participants to move and explore the environment. This factor is crucial considering the role of proprioceptive feedback and the vestibular system in the encoding and maintenance of spatial configurations in memory.” From your manuscript, it remains unclear to what extent the participants in your task are provided with proprioceptive and vestibular feedback. In the abstract you mention a restriction to rotational exploration. In the introduction, you mention a movable chair (very limited proprioceptive/vestibular feedback). In your ‘Environment Navigation/Locomotion’ description it sounds like participants can use continuous artificial locomotion, which also does not provide proprioceptive or vestibular feedback. If you want to ensure proprioception and vestibular feedback, participants should be able to move towards the objects using natural (room scale) locomotion. The moveable chair is not mentioned in the methods section.

 P3: Why do you use the RAVLT test if you do not expect it to correlate with the VSCT?

 P4: What sociodemographic data will be collected and why? Only data that is linked to a specified hypothesis should be collected.

 P5: You say that you chose household objects to enhance ecological relevance. However, I am not sure how ecologically relevant these objects are in the highly artificial empty environment that is used in the task. A better argument would be the general familiarity with specific object types.

 P5: You removed or controlled the prominent spatial cues that could be used for orientation (sun position, shadows, floor texture), which is very important for this task. However, you should also consider creating an own skybox. It is not easily visible, but the standard skybox in Unity does contain directional differences. The light on the horizon is slightly brighter in some directions and less bright in other directions.

 P5: “We select three objects for each scene: two objects near each other and one facing them” Do you mean that the third object is on the opposite side (in the back of the participant if he/she is looking at the two objects near each other)?

 P5: Training phase: What exact instructions will be provided to the participants? What is the task and exact purpose?

 P5: The description of the locomotion method(s) requires improvements. Is this a combination of room scale (natural locomotion) and controller-based (artificial) locomotion? If yes, how large is the play space? How fast is the continuous artificial locomotion? Can participants select an artificial locomotion speed between zero and the maximum speed or is it either zero or maximum speed? Why do you write in the abstract that navigation is restricted to rotational exploration if you describe movement here? What are the exact instructions provided to the participants concerning locomotion?

 P5: Figure 1 could be improved. I cannot identify the rug on the floor described in the text. Are the grey circles supposed to be rugs? What is the washed out smudge in the middle of .c and .d?

 P6: AV and VR should be fully written out when they are mentioned for the first time.

 P6: You do not motivate the investigation of the variables age and sex. This should be based on cited previous findings and formulated hypotheses.

 P8: “Ethical approval has already been granted by the relevant ethical committee” To which ethics committee was the research protocol submitted? You describe it in the text above, but it should also be mentioned here.

 P8: Consent for publication: Please describe how you will ensure that no individual data will be included. How will the data be anonymized?

 Taken together, although the proposed task may not as innovative as the authors describe it (similar approaches have already been used), it may be a meaningful approach to assess spatial memory if the limitations mentioned above are addressed.

Is the study design appropriate for the research question?

Partly

Is the rationale for, and objectives of, the study clearly described?

Partly

Are sufficient details of the methods provided to allow replication by others?

No

Are the datasets clearly presented in a useable and accessible format?

Not applicable

Reviewer Expertise:

Spatial cognition, spatial memory, landmarks, virtual reality

I confirm that I have read this submission and believe that I have an appropriate level of expertise to confirm that it is of an acceptable scientific standard, however I have significant reservations, as outlined above.

# Response

**Published:** 2025-04-26

**Authors:** Giorgio Li Pira

**Affiliations:** Psicologia, Universita degli Studi di Bologna Dipartimento di Psicologia Renzo Canestrari, Bologna, Emilia-Romagna, Italy

P3: “Furthermore, 2D tests do not allow participants to move and explore the environment. This factor is crucial considering the role of proprioceptive feedback and the vestibular system in the encoding and maintenance of spatial configurations in memory.” From your manuscript, it remains unclear to what extent the participants in your task are provided with proprioceptive and vestibular feedback. In the abstract you mention a restriction to rotational exploration. In the introduction, you mention a movable chair (very limited proprioceptive/vestibular feedback). In your ‘Environment Navigation/Locomotion’ description it sounds like participants can use continuous artificial locomotion, which also does not provide proprioceptive or vestibular feedback. If you want to ensure proprioception and vestibular feedback, participants should be able to move towards the objects using natural (room scale) locomotion. The moveable chair is not mentioned in the methods section.  Thank you for this important observation. We agree that the level of proprioceptive and vestibular input must be clearly specified. As correctly noted, translational movement is not performed by participants in our study. Instead, participants are seated in a swivel chair that allows them to actively rotate their torso left and right, enabling 360-degree exploration of the environment. These self-generated rotations provide limited but present vestibular and proprioceptive feedback. In particular, yaw-plane movements engage the vestibular system via stimulation of the semicircular canals responsible for detecting angular acceleration (Ehtemam et al., 2012), while proprioceptive input is involved through trunk muscle activity and postural adjustments during chair rotation (Ivanenko et al., 1999). We have updated the text to better specify at page 3  We would also like to clarify that the term “locomotion” is no longer used in the manuscript, except as part of a previous section heading, which has now been renamed to “Exploration” to avoid misunderstanding. As the reviewer correctly points out, natural, translational locomotion is typically necessary to provide robust proprioceptive and vestibular input. However, our approach was deliberately chosen to offer at least partial sensory feedback while maintaining compatibility with a wider range of physical environments.  This design makes the software accessible to users who may not have access to large, open spaces required for room-scale locomotion, while still allowing for active sensorimotor exploration through torso rotations. We have now clarified this distinction in the abstract, methods, and discussion sections and emphasized this as a design trade-off that balances ecological validity with practical usability. We also acknowledge that future iterations of the task may integrate full-body locomotion to further enhance sensory realism (Campos et al., 2010); (Chrastil & Warren, 2013).   

 P3: Why do you use the RAVLT test if you do not expect it to correlate with the VSCT? 

 Thank you for this important question. We included the Rey Auditory Verbal Learning Test (RAVLT) in our study not because we expected it to correlate with the Visual Spatial Configuration Task (VSCT), but precisely to assess discriminant validity. The RAVLT measures verbal episodic memory, while the VSCT targets visuospatial configuration memory—two constructs that are theoretically and empirically distinct.  By including the RAVLT and confirming that it does not significantly correlate with performance on the VSCT, we provide evidence that the VSCT measures the specific visuospatial component of the memory. We have updated the text in accordance with P4.   

 P4: What sociodemographic data will be collected and why? Only data that is linked to a specified hypothesis should be collected.  Thank you for this observation. We collected age and gender as sociodemographic variables because both have been shown to influence spatial navigation abilities in previous research (Moffat et al., 2007); (Newhouse et al., 2007). Our primary reason for collecting these variables was to ensure that our control and experimental groups were comparable on key factors that could confound task performance. Although we did not formulate specific hypotheses regarding age or gender effects, their inclusion allows us to confirm group equivalence and strengthen the internal validity of our findings. 

 The text has been corrected to clarify on page 9 

 P5: You say that you chose household objects to enhance ecological relevance. However, I am not sure how ecologically relevant these objects are in the highly artificial empty environment that is used in the task. A better argument would be the general familiarity with specific object types.  Thank you for this helpful suggestion. We agree with the reviewer that, given the controlled nature of the virtual environment, referring to the use of household objects as enhancing "ecological relevance" may have been misleading. Instead, we have revised the text to emphasize that these objects were chosen for their high familiarity and recognizability, which helps reduce biases due to the novelty of the object, encoding variability, and supports more consistent performance across participants. This change has been reflected in the revised manuscript accordingly at P7. 

 P5: You removed or controlled the prominent spatial cues that could be used for orientation (sun position, shadows, floor texture), which is very important for this task. However, you should also consider creating an own skybox. It is not easily visible, but the standard skybox in Unity does contain directional differences. The light on the horizon is slightly brighter in some directions and less bright in other directions.  Thank you for this valuable observation. We are grateful for you pointing out the potential directional cues present in Unity's default skybox. To minimize the influence of such unintentional orientation cues, we specifically modified the sun position to be directly overhead, simulating a noon-like lighting condition. This adjustment ensures that light distribution remains uniform across all directions, thereby preventing participants from using subtle variations in brightness as a spatial reference. We have now clarified this detail in the manuscript.   

 P5: “We select three objects for each scene: two objects near each other and one facing them” Do you mean that the third object is on the opposite side (in the back of the participant if he/she is looking at the two objects near each other)? 

 Thank you for the question and for allowing us to clarify. Yes, you are absolutely right—the third object is positioned on the opposite side, facing the pair of nearby objects. In other words, if the participant is oriented toward the two adjacent objects, the third object would be located behind them, forming a triangular spatial configuration. We have updated the manuscript to clarify this spatial arrangement at p7   

 P5: Training phase: What exact instructions will be provided to the participants? What is the task and exact purpose?  Thank you for this important point. We have now uploaded the full instructions provided to participants in the Supplementary Material for full transparency. As specified in the manuscript, participants were simply told that they would later be asked to correctly identify the spatial position of each object they had seen during the training phase. No additional information about the specific structure or aim of the task was given, in order to avoid biasing their encoding strategy and to maintain consistency across participants. We have also clarified in the revised manuscript that the task has been modified and no longer includes a separate training phase; instead, participants begin responding to spatial location questions from the outset and learn through trial and error on p7. 

 P5: The description of the locomotion method(s) requires improvements. Is this a combination of room scale (natural locomotion) and controller-based (artificial) locomotion? If yes, how large is the play space? How fast is the continuous artificial locomotion? Can participants select an artificial locomotion speed between zero and the maximum speed or is it either zero or maximum speed? Why do you write in the abstract that navigation is restricted to rotational exploration if you describe movement here? What are the exact instructions provided to the participants concerning locomotion?  Thank you for these detailed questions and the opportunity to clarify.  In our study, controller-based (artificial) locomotion was intentionally avoided in order to minimize the risk of motion sickness, especially given the stationary setup. Participants were instructed to explore the environment exclusively through rotational movement, using a swivel chair that allowed them to rotate their torso and head to view the full 360-degree virtual space.  We acknowledge that referring to "navigation" may have caused confounding interpretations. To avoid such confusion, we updated the terminology throughout the manuscript—especially in the abstract—to specify that the task involves rotational exploration only. While participants do not engage in translational locomotion, we do consider rotational movement to be a form of movement in its own right, as it activates both the vestibular system and the musculoskeletal system, particularly involving torso and leg muscles during active chair rotation (Ivanenko et al., 1999).  Regarding participant instructions: they were explicitly told to explore the environment by rotating the chair, and no other movement methods were allowed. The exact wording of the instructions has been included in the Supplementary Materials for full transparency.  We have revised the relevant sections in the abstract and methods to better reflect this setup. 

 P5: Figure 1 could be improved. I cannot identify the rug on the floor described in the text. Are the grey circles supposed to be rugs? What is the washed out smudge in the middle of .c and .d? 

 Yes, you are correct—the grey circles are intended to represent the rugs mentioned in the text. We have clarified this by adding a specification in the figure caption to make this clearer to readers. Additionally, we acknowledge that the image quality appears to have been downgraded after submission, which may have contributed to the lack of clarity, particularly the washed-out appearance in panels c and d. To address this, we will provide a higher-resolution version of Figure 1 to ensure all visual elements are clearly visible and accurately represented in the final publication. 

 P6: AV and VR should be fully written out when they are mentioned for the first time.  Thank you for pointing this out. You are absolutely right—"VR" was fully written out on the first mention, while "AV" was introduced later without explanation. We have now corrected this oversight by fully writing out "auditory verbal (AV)" at its first mention in the manuscript to ensure clarity and consistency. 

 P6: You do not motivate the investigation of the variables age and sex. This should be based on cited previous findings and formulated hypotheses. 

 Thank you for this observation. We have now addressed this point by adding a clear rationale and relevant citations in the manuscript. Specifically, we note that age and sex have been shown to influence spatial navigation performance, with older adults often exhibiting declines in spatial memory and orientation abilities, and sex differences being reported in strategy use and performance levels (Moffat et al., 2007); (Newhouse et al., 2007).  While we did not formulate specific hypotheses regarding age and sex effects on task performance, these variables were collected to allow us to control for potential group differences and to explore their influence in an exploratory manner. This addition has been integrated into the revised manuscript.  P8: “Ethical approval has already been granted by the relevant ethical committee” To which ethics committee was the research protocol submitted? You describe it in the text above, but it should also be mentioned here.  This has now been addressed by explicitly stating the name of the ethics committee in the relevant sentence. The revised sentence now reads: 

 “Ethical approval has already been granted by the [Name of Committee] Ethics Committee,” in alignment with the description provided earlier in the manuscript. 

 P8: Consent for publication: Please describe how you will ensure that no individual data will be included. How will the data be anonymized? 

 Thank you for the comment. We have now addressed this point by adding a description to the Methods section specifying that all data collected will be fully anonymized prior to analysis and publication. No identifying personal information (e.g., names, contact details, or video recordings) will be collected or stored. Participants are identified only by randomized numeric codes, and all reported results will be presented at the group level, ensuring that no individual can be identified in any publication or presentation of the data. 

   Taken together, although the proposed task may not as innovative as the authors describe it (similar approaches have already been used), it may be a meaningful approach to assess spatial memory if the limitations mentioned above are addressed.
